# Refining sequence-to-expression modelling with chromatin accessibility

**DOI:** 10.1093/bioinformatics/btag199

**Published:** 2026-04-26

**Authors:** Orsolya Lapohos, Gregory J Fonseca, Amin Emad

**Affiliations:** Department of Quantitative Life Sciences, McGill University, Montreal, Quebec, H3A 0G4, Canada; Meakins-Christie Laboratories, Research Institute of the McGill University Health Centre, Montreal, Quebec, H4A 3J1, Canada; Mila, Quebec AI Institute, Montreal, H2S 3H1, Canada; Meakins-Christie Laboratories, Research Institute of the McGill University Health Centre, Montreal, Quebec, H4A 3J1, Canada; Department of Medical Sciences, Khalifa University, Abu Dhabi, 127788, United Arab Emirates; Department of Quantitative Life Sciences, McGill University, Montreal, Quebec, H3A 0G4, Canada; Meakins-Christie Laboratories, Research Institute of the McGill University Health Centre, Montreal, Quebec, H4A 3J1, Canada; Mila, Quebec AI Institute, Montreal, H2S 3H1, Canada; Department of Electrical and Computer Engineering, McGill University, Montreal, Quebec, H3A 0G4, Canada; The Rosalind and Morris Goodman Cancer Institute, Montreal, Quebec, H3A 1A3, Canada

## Abstract

**Motivation:**

Sequence-to-expression models typically do not consider chromatin accessibility, a major factor limiting gene regulation. We hypothesized that supplying accessibility as an input feature would allow a sequence-to-expression model to focus on important open regions of the genome.

**Results:**

We found that the performance of such an augmented model was significantly better than that of sequence-only or accessibility-only models with similar architectures. Specifically, its ability to predict the expression of highly variable genes and gene expression in other cell types improved, and higher attribution scores in the input DNA sequences of the augmented model conformed to accessibility, enabling the learning of cell type-specific sequence patterns. Additionally, we show that fine-tuning a pre-trained sequence-only model with both sequence and accessibility can boost performance further and highlight the importance of sequencing depth in sequence-to-expression prediction.

**Availability and Implementation:**

Source code is available on GitHub at https://github.com/lapohosorsolya/accessible_seq2exp.

## 1 Introduction

Metazoan gene expression is a process that is temporally and spatially orchestrated by a multitude of factors. At the lowest level, the blueprint for gene expression is ciphered into non-coding regulatory regions, defining the universe of DNA-binding proteins that direct gene regulation (Alberts et al. 2002). Although distal non-coding regions such as enhancers play important roles in controlling gene expression (FitsGerald et al. 2004, Smith et al. 2023), the promoter is arguably the master regulator. At the next level, the set of possible regulatory interactions is limited by context—chromatin accessibility and the set of available DNA-binding proteins both depend on cell type and environment (Heinz et al. 2015). Available regulatory proteins compete and cooperate to bind accessible regions and ultimately activate or pause transcription initiation (Heinz et al. 2010, Fonseca et al. 2019). Gene expression is further regulated during transcription and translation (Alberts et al. 2002).

Despite the myriad of interactions regulating gene expression, sequence-to-expression models have been successful in predicting transcriptional activity from only promoter sequences (Zhou et al. 2018, Agarwal and Shendure 2020, Kelley 2020, Li et al. 2022, Hepkema et al. 2023, Michielsen et al. 2024). The 1D convolutional neural network (CNN) is naturally suited to the task of learning local sequence patterns and is the most frequently used base architecture for sequence-to-expression modelling, but modifications and new architectures (such as attention mechanism) have been added to recent models in order to improve performance (Avsec et al. 2021, Linder et al. 2025). However, most existing models do not make use of rate-limiting gene-regulatory factors, such as chromatin accessibility, in order to predict gene expression.

Several sequence-to-expression models have incorporated chromatin accessibility *implicitly* by simultaneously learning to predict gene expression and epigenomic tracks, with the expectation that this multi-task formulation would capture biological features and mechanisms shared between the outputs (Kelley et al. 2018, Avsec et al. 2021, Karbalayghareh et al. 2022). On the other hand, previous work incorporating chromatin accessibility *explicitly* as input to sequence-based models has largely been limited to transcription factor (TF) binding prediction. For example, Srivastava et al. (2021) used a bimodal CNN-LSTM (long short-term memory) architecture to embed sequence and chromatin predeterminants into a shared latent space before predicting TF binding. Using a more direct approach, Arora et al. (2023) jointly modelled TF binding using a CNN architecture with additional chromatin state tracks supplied as input channels alongside one-hot-encoded DNA sequences. To model gene expression, Javed et al. (2025) first pre-trained a multi-modal transformer on a masked chromatin accessibility prediction task and then fine-tuned it on a gene expression prediction task. Lin et al. (2024) had a different approach to this problem, encoding promoter and nearby enhancer sequences using a CNN before incorporating additional contextual information (such as accessibility) in an “interaction encoder” to predict gene expression. However, to the best of our knowledge, a simpler approach supplying a chromatin accessibility track as an additional CNN input channel has not been studied specifically in the context of gene expression prediction.

The goal of this study is to explore this model augmentation strategy, which is compatible with virtually all sequence-to-expression architectures. Accordingly, we designed a proof-of-concept study to thoroughly evaluate the effect of augmenting a sequence-to-expression model using chromatin accessibility as an input feature. For computational tractability, we selected a model with relatively few parameters: we opted to use a CNN-based architecture similar to Xpresso (Agarwal and Shendure 2020), rather than a transformer-based model, and to limit input sequence lengths to the 2 kb regions flanking the transcription start sites of protein-coding genes. By doing so, we were able to train, evaluate, and interpret accessibility-augmented and ablated architectures on 12 different cell types from three independent human multiome RNA- and ATAC-seq datasets with nested cross-validation, as well as perform additional scrambling experiments.

The contributions of this study are as follows. We showed that our accessibility-augmented model predicted the expression of held-out genes more accurately than models with similar architectures that only used sequence information or accessibility. This strategy also resulted in a greater improvement on the difficult task of highly variable gene expression prediction, while being able to generalize to other cell types. This analysis was enabled by our use of single-cell data, which revealed highly variable genes in each dataset. Additionally, models that used scrambled inputs performed significantly worse compared to our augmented model, revealing that all input data modalities contributed to the prediction performance. Importantly, we also showed that attribution scores obtained via the Shapley Additive Explanations DeepExplainer (Lundberg and Lee 2017) in all DNA input channels became more correlated with accessibility. By analyzing *k*-mer attribution scores and known motifs, we additionally showed that the augmented model was less reliant on CpG content, and that cell type-specific sequence patterns were better modelled when ATAC-seq was included. Finally, we demonstrated that a pre-training strategy could further boost performance and refine attribution scores. The strategies here can be adapted to other sequence-to-expression models with ease, potentially benefiting various downstream tasks.

## 2 Methods

### 2.1 Promoter sequences

The genome sequence FASTA and comprehensive gene annotation GTF files for the GRCh38.p14 reference genome (release 44) were downloaded from GENCODE (Frankish et al. 2023). Then, the annotation file was filtered to obtain the coordinates of “Ensembl canonical” transcripts for protein-coding genes, which were used to extract the coding strand sequence 1 kb upstream and 1 kb downstream of the start of the first exon (transcription start site, or TSS).

### 2.2 Datasets

Human single-nucleus multiome assay for transposase-accessible chromatin (ATAC) and gene expression datasets for peripheral blood mononuclear cells (PBMC), brain, and jejunum, as well as a human PBMC single-cell gene expression dataset with cell surface marker antibody-derived tags (ADTs) were sourced from 10× Genomics (Data availability). Dataset details are listed in Table S1. Preprocessing and annotation of multiome and single-cell datasets are described in Notes S1 and S2, respectively. Four major cell types were used from each dataset, shown in Fig. S1A–C. Cell type marker genes and numbers are indicated in Tables S2 and S3, respectively.

### 2.3 Representing chromatin accessibility and gene expression for sequence-to-expression modelling

To obtain pooled cell type-specific ATAC tracks for each 2 kb input sequence around the TSS, raw ATAC-seq fragments mapped to the cell type of interest were aligned to input sequence coordinates. These ATAC tracks were smoothed using the 1D Gaussian filter function from the SciPy package (with standard deviation 20) (Virtanen et al. 2020) since the raw tracks were noisy, and Gaussian smoothing yielded appropriate scale-space representations for the task at hand. Track values were then min-max normalized by cell type. For other analyses, a summary metric termed “auATAC” was also defined to represent the accessibility of a gene promoter as the area under its normalized 2 kb ATAC track.

To obtain a measure of gene expression by cell type, the proportion of cells of the cell type of interest with a nonzero unique molecular identifier (UMI) count was calculated for each gene. This metric, termed “GEx” in this study, represents the probability that a gene is expressed while ensuring that the output values range between 0 and 1. We opted to use gene expression probabilities rather than transcript abundance for two reasons. First, a probabilistic representation may be less biased by enhancer-mediated and post-transcriptional regulation of mRNA levels. Second, probabilities provide a more comparable measure for evaluation metrics and for cross-cell type prediction.

To analyze the similarity of gene expression or accessibility between cell types, the Pearson correlation coefficient between all GEx or auATAC values was calculated (Fig. S1D–F). Spearman correlation was used to determine concordance between GEx and auATAC (Fig. S1G–I).

### 2.4 Model architecture

**Figure 1:**
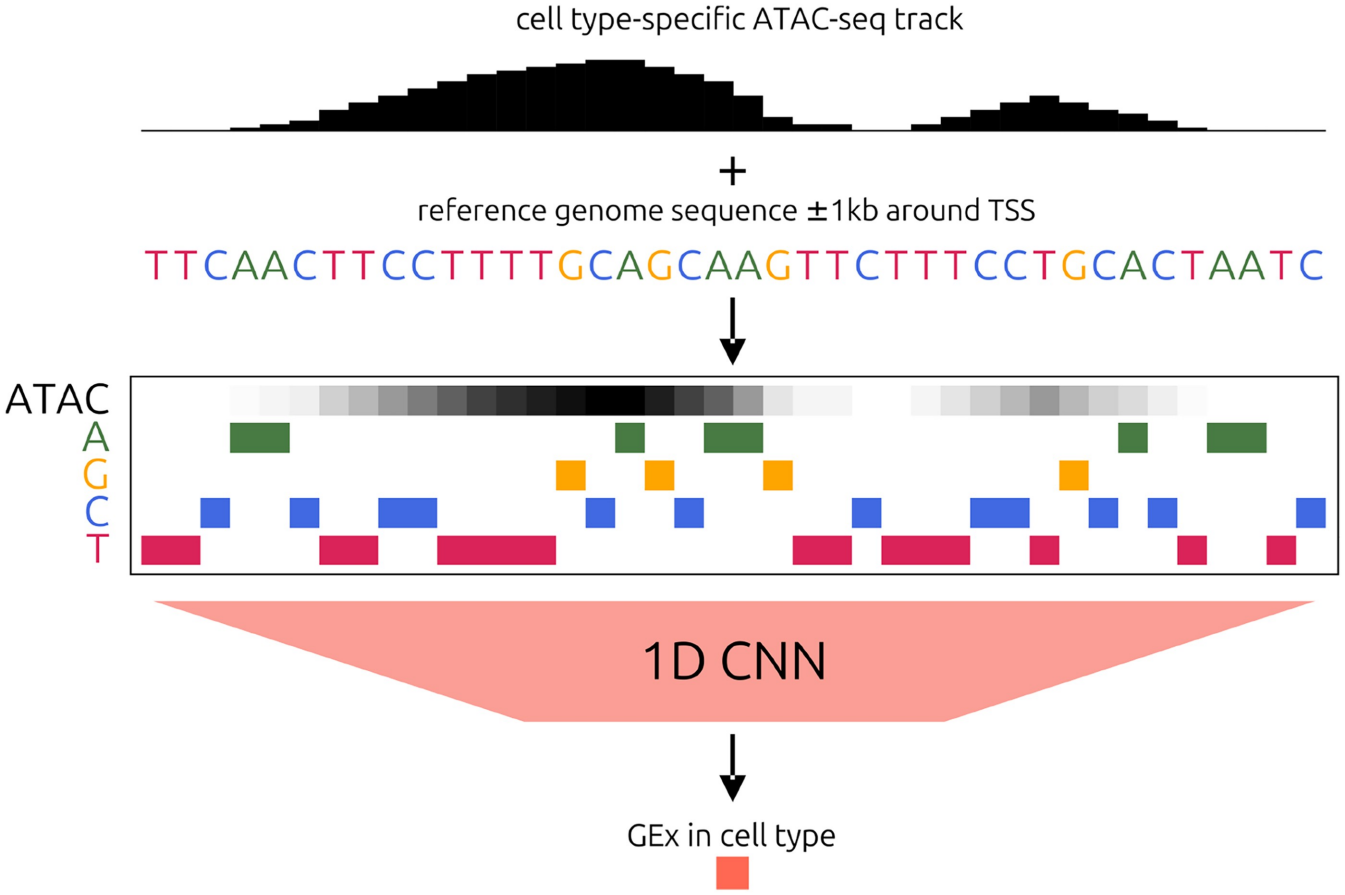
Overview of the ATAC-augmented sequence-to-expression model. Each input example represents the promoter of a gene with an ATAC-seq track for a specific cell type. The 1D CNN outputs a single value that represents the proportion of cells expressing the input gene.

Figure 1 shows an overview of our augmentation strategy which uses context-specific chromatin accessibility as an input feature to a sequence-to-expression model. One-hot-encoded DNA sequences with pooled and normalized ATAC-seq tracks serve as the five input channels to the neural network. The model outputs a single value that represents the expression of the input gene. The architecture of the neural network is based on Xpresso (Agarwal and Shendure 2020), but with a shorter input sequence (2 kb instead of 10 kb), an additional input channel, fixed depth and layer widths, and an additional dense layer (details in Table S4 and Fig. S2). Our model was constructed with PyTorch v2.2 (Paszke et al. 2019). For ablation experiments, model architecture remained unchanged, other than the input channels, which could consist of the cell type-specific ATAC track (1 input channel; 3 640 193 total parameters), the one-hot-encoded DNA sequence of the promoter (4 input channels; 3 642 497 total parameters), or both (5 input channels; 3 643 265 total parameters).

### 2.5 Model training

Each model was trained and tested using five-fold cross-validation (CV) on the entire set of human autosomal protein-coding genes (19 095), where each test set consisted of unique genes. Training for each fold was repeated five times, each with different random seeds used for model initialization and splitting inner training and validation sets. Gene order was preserved across all datasets for each seed in each CV fold. The model was trained with mean squared error (MSE) loss, defined as $L=\frac{1}{n}\sum_{i=1}^{n}{\left(y_{i}-\hat{y_{i}}\right)}^{2}$ where *n* is the number of genes, $y_{i}$ is the ground truth GEx of gene *i*, and $\hat{y_{i}}$ is the predicted GEx for gene *i*. Training was guided by the Adam optimizer (Kingma and Ba 2017), with learning rate $5\times 10^{-5}$ and weight decay set to $10^{-3}$. Batch size was set to 512, and the training procedure was run for 500 epochs. Validation loss was computed every four epochs, and the model with the lowest validation loss was saved.

### 2.6 Fine-tuning of pre-trained models

An additional model training strategy involved a two-step procedure. First, a sequence-to-expression model was trained on sequence only. Then, these optimized weights were used to initialize a new full model, with input ATAC channel weights initialized randomly. This full model was then fine-tuned by training on both DNA and ATAC features. The fine-tuning procedure was performed for a maximum of 200 epochs, with reduced weight decay ($10^{-4}$), a lower learning rate ($10^{-5}$), and using the same five-fold CV and random seeds.

### 2.7 Evaluation

For each experiment, performance was assessed across five folds. For each fold, the average prediction across five models trained with different random seeds was evaluated. Reported means and standard deviations were calculated across the five folds. Four different metrics were calculated: Pearson correlation coefficient, Spearman correlation coefficient, MSE, and the coefficient of determination ($R^{2}$).

### 2.8 Naïve prediction and evaluation

For each input gene promoter, the naïve predictor outputs its auATAC. Evaluation was performed using the test sets from the same five-fold CV, calculating the Spearman correlation coefficient between the prediction (auATAC) and the ground truth GEx.

### 2.9 *Post hoc* model explanations

We obtained attribution scores for each held-out test sample using the SHapley Additive exPlanations (SHAP) DeepExplainer (Lundberg and Lee 2017). As background for each sample, we supplied 100 shuffled versions that preserved its dinucleotide frequencies, using the dinucleotide shuffler from the DeepLIFT package (Shrikumar et al. 2019). For background samples used to explain the DNA+ATAC model, values in the ATAC input channel were randomly shuffled as well. To summarize positional attribution scores by input channel, the mean SHAP score for each sequence position in each channel was calculated across five random seeds, for each CV fold. Then, means from all held-out test genes of the five-fold CV were concatenated. Finally, the mean SHAP score was calculated at each position across all held-out test genes, for each input channel.

### 2.10 *k*-mer attribution analysis

We obtained attribution scores for *k*-mers with k=2 and 6, where the score of a single *k*-mer instance in an input sequence is the mean SHAP score across the corresponding bases at each position. For every possible *k*-mer, we obtained scores at all instances within the input sequences, for all 25 trained models (five CV folds and five random seeds), for one cell type. For k=2, we took the mean attribution score across all instances and averaged these again across cell types to obtain an overall cell type-agnostic score for each dinucleotide. Then, we ranked 2-mers using these scores to compare model types. To analyze longer sequence patterns that may confer some cell type-specificity, we looked at 6-mers. For each 6-mer, we took the average across all its instances with positive SHAP scores to determine its role in positive regulation of gene expression in isolation. Then, we ranked all 6-mers for each cell type and compared the top 10% pairwise using the Jaccard index, defined as |A∩B|/|A∪B| for two sets *A* and *B*. We also repeated this analysis using all instances, regardless of score sign.

### 2.11 Motif discovery

To obtain important motifs for each cell type-specific model, we ran TF-MoDISco (Shrikumar et al. 2020) (parameters: max seqlets 10 000, window size 2000, seqlet core size 18, and trim size 24) on SHAP scores of all genes with mean auATAC greater than the mean for that cell type. Then, Tomtom (Gupta et al. 2007) was used to match the resulting positive patterns to known motifs from a non-redundant motif set generated with AmalgaMo (Lapohos and Fonseca 2026) with HOCOMOCO v12 human motifs (Vorontsov et al. 2024) as input.

### 2.12 Promoter variant effect prediction

Using the GTEx eQTL promoter variant effect benchmark dataset curated by Jaganathan et al. (2025), we tested whether the change in predicted GEx between the reference and alternative sequences had the correct sign. We calculated the area under the receiver operating characteristic curve (auROC) for the classification of variants causing over- versus under-expression, as well as under-expression versus no effect, and no effect versus over-expression. For each CV fold, auROC was calculated for promoter variants corresponding to held-out promoters only.

### 2.13 Experiments using longer sequences

To ensure there would be no overlap between longer input sequences, we defined a new train-test split using entire chromosomes, holding out promoter regions in chromosomes 2 and 9 as the single test set. Then, we trained similar augmented and ablated model architectures using five different input sequence lengths ranging from 1.5 to 13.5 kb flanking the TSS (see Table S5 for details). We repeated this process across five model replicates (random seeds), keeping the same hyperparameters as those described in the Model Training section, with three exceptions. The first exception was convolutional block width, which necessarily increased with input length. The second exception was batch size, which was reduced for longer sequences due to compute constraints. Lastly, the models were trained for a maximum of 100 epochs.

### 2.14 Statistical testing

The sets of held-out test genes used for evaluation across all five folds were preserved across all models. Thus, we used the one-sided Wilcoxon signed-rank test [from SciPy (Virtanen et al. 2020)] to test for statistical significance, when comparing performance of different models.

## 3 Results

### 3.1 ATAC-seq refined sequence-to-expression prediction

In order to determine whether providing chromatin accessibility as an additional input feature could improve sequence-to-expression modelling, we augmented a vanilla CNN-based model architecture by including an extra input channel that represents a pooled ATAC-seq track for a specific cell type, in addition to the one-hot encoding of the input sequence (Fig. 1). This architecture could be ablated easily by modifying only the set of input channels. We trained such models on sequence (DNA-only), accessibility (ATAC-only), and both (DNA+ATAC), to predict the proportion of cells within a cell type that express a gene (GEx).

First, we assessed DNA-only as our baseline sequence-to-expression model. This model achieved an average Pearson correlation of 0.3658 across the four major cell types in the PBMC dataset (Table 1, DNA only). Spearman correlations were higher for all four cell types, with an average of 0.5319. Performance was better on the brain and jejunum datasets overall, with average Pearson correlations of 0.4966 and 0.4573, and Spearman correlations of 0.5970 and 0.5620, respectively (Table S6). The same trend was observed with MSE and $R^{2}$ metrics.

**Table 1:** Results of ablation experiments with the PBMC dataset.

Cell type	Model	Pearson *r*	Spearman *r*
B cell	DNA only	0.362 ± 0.020	0.534 ± 0.006
	ATAC only	0.474 ± 0.016	0.704 ± 0.006
	**DNA+ATAC**	**0.522** ± **0.016**	**0.720** ± **0.006**
CD14${\;}^{+}$ monocyte	DNA only	0.384 ± 0.013	0.524 ± 0.011
	ATAC only	0.547 ± 0.009	0.734 ± 0.008
	**DNA+ATAC**	**0.580** ± **0.010**	**0.742** ± **0.008**
CD4 T cell	DNA only	0.354 ± 0.017	0.530 ± 0.008
	ATAC only	0.475 ± 0.008	0.713 ± 0.008
	**DNA+ATAC**	**0.512** ± **0.009**	**0.717** ± **0.005**
CD8 T cell	DNA only	0.364 ± 0.019	0.539 ± 0.008
	ATAC only	0.476 ± 0.010	0.707 ± 0.006
	**DNA+ATAC**	**0.522** ± **0.012**	**0.717** ± **0.005**

Performance metrics on the held-out test sets are shown. All comparisons within cell type are significantly different by one-sided Wilcoxon signed-rank test (*P* < 0.05), except ATAC only versus DNA+ATAC Spearman *r* for CD4 T cell.

As expected, ATAC alone was a better predictor of gene expression than sequence alone (Table 1, ATAC only). This was already apparent in a naïve predictor that predicts GEx based on its auATAC (Methods). The average Spearman correlation of the naïve predictions with ground truth GEx for PBMCs was 0.694 (Fig. S3). The ATAC-only model achieved an average Pearson correlation of 0.4930 and Spearman correlation of 0.7145 on the PBMC dataset, improving on the naïve predictor.

Combining DNA with ATAC significantly and consistently improved model performance across all cell types (Table 1, DNA+ATAC; Table S6; p<0.05 based on one-sided Wilcoxon signed-rank tests, comparing DNA+ATAC to ablated models). The average Pearson and Spearman correlations for PBMCs were 0.5339 and 0.7241, for the brain dataset, they were 0.6621 and 0.7579, and for the jejunum dataset 0.5522 and 0.6925.

**Figure 2:**
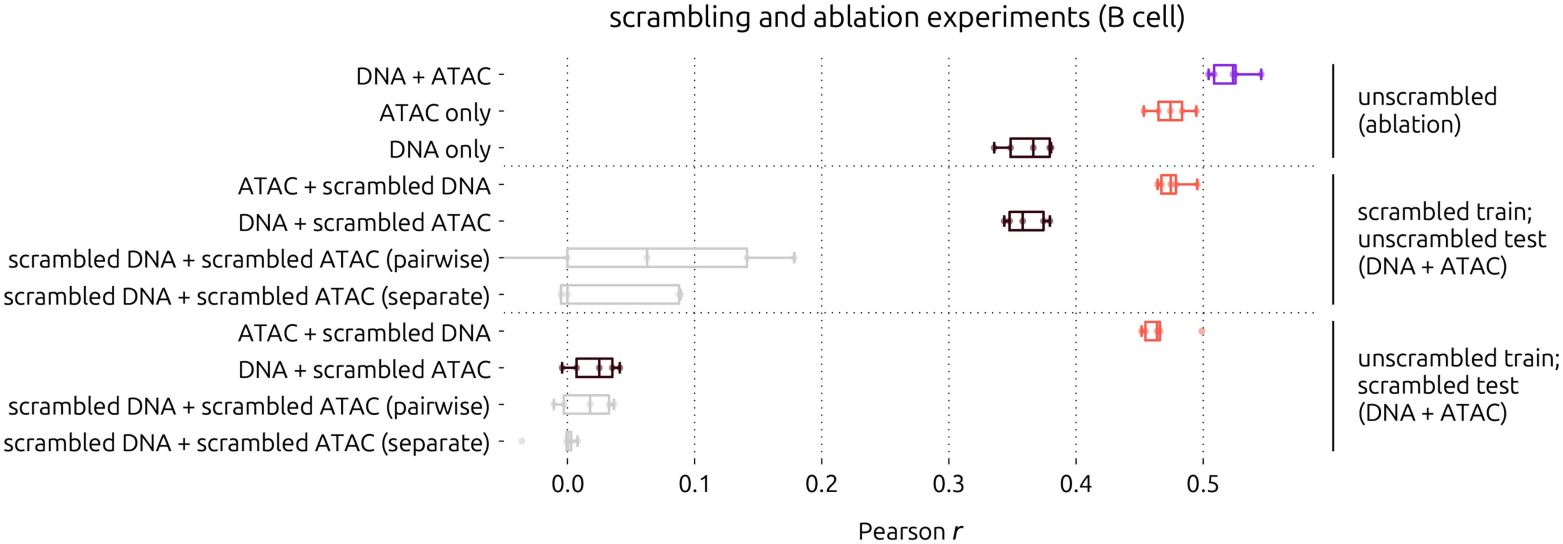
Scrambling experiments with B cell models. Pearson correlations of predicted versus ground truth GEx across five-fold CV for each indicated ablated or scrambled model. Pairwise scrambling means the promoter sequence and ATAC track were kept together. Separate scrambling means promoter sequences, and ATAC tracks were scrambled independently. All vertically adjacent pairs of boxplots, except for the bottom pair, are significantly different by one-sided Wilcoxon signed-rank test (*P* < 0.05).

Since ATAC alone is a relatively strong predictor of GEx, we performed scrambling experiments to determine the contribution of DNA sequence to the DNA+ATAC model output. We scrambled one or both input features in the training sets with respect to the target genes, trained new models, and evaluated their performance on the unscrambled test sets. We also re-evaluated predictions of models trained on unscrambled training sets using scrambled test sets as ground truth. In all scrambling experiments, performance was significantly worse than that of the unscrambled DNA+ATAC model (Fig. 2; *P* < 0.05 based on one-sided Wilcoxon signed-rank tests).

When the DNA inputs in the training set were scrambled, the DNA+ATAC model achieved approximately the same performance on the unscrambled test set as the unscrambled ablated ATAC-only model (mean Pearson r≈0.47), indicating that unscrambled DNA is required to achieve the full performance of the DNA+ATAC model. Likewise, when the ATAC inputs in the training set were scrambled, similar performance was achieved on the unscrambled test set as the ablated DNA-only model (mean Pearson r≈0.36). We also experimented with scrambling both inputs for training, to determine whether combinatorial effects between sequence patterns and accessibility could be learned by the augmented model. When inputs were scrambled pairwise (i.e. DNA and ATAC of the same gene promoter were kept together) the model performed poorly, with mean Pearson r=0.0492. When inputs were scrambled separately (independently with respect to the target gene), performance was significantly worse (*P*=0.0339, one-sided Wilcoxon signed-rank test), with mean Pearson r=−0.0317. This suggests that, despite training on scrambled data, some predictive patterns can be learned when DNA and ATAC are complementary. Our findings were corroborated by a similar experiment where only the test set was scrambled (Fig. 2). Altogether, results of the ablation and scrambling experiments showed that the input DNA sequences contributed meaningfully to the DNA+ATAC model output.

### 3.2 ATAC-seq feature improved cross-cell type and highly variable gene expression prediction

We evaluated generalizability by testing each trained cell type-restricted model on other cell types within the same dataset. In all three datasets, Pearson correlations on the cross-cell type prediction tasks were similar to those obtained when predicting on the same cell type, for both the ablated and augmented models (Fig. S4). These results indicate that the ATAC-seq input refined sequence-to-expression prediction from a generalization perspective as well. However, it should be noted that the Pearson correlation coefficients between ground truth GEx of different cell types within the same dataset ranged from 0.699 to 0.981 (Fig. S1D–F, top). Cross cell-type Pearson correlation of auATAC was higher, with values between 0.817 and 0.992 (Fig. S1D–F, bottom). Thus, it is not surprising that sequence-to-expression models could generalize between cell types.

**Figure 3:**
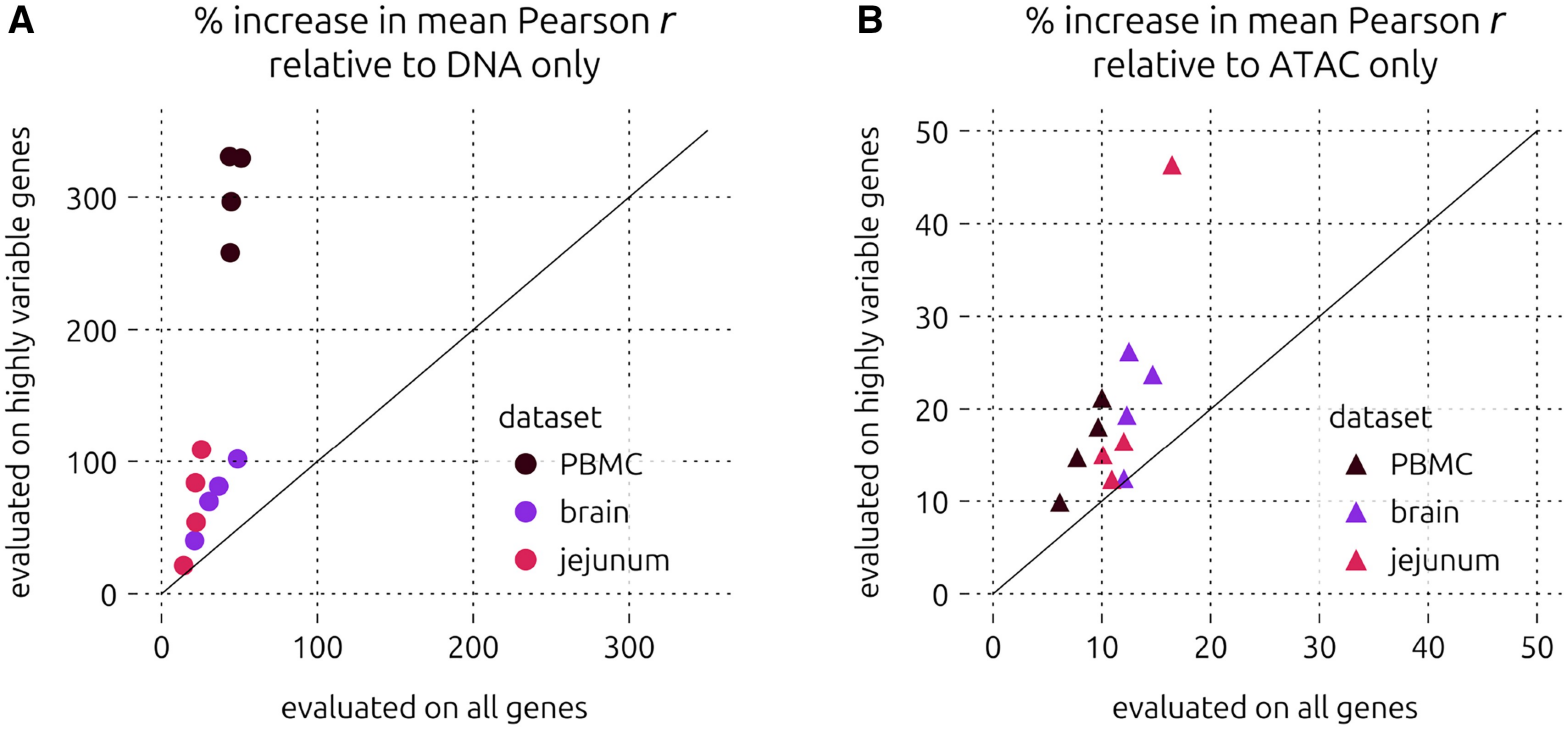
Highly variable gene expression prediction results. (A) Percent increase in mean Pearson correlation from DNA only to DNA+ATAC for all major cell types in all three multiome datasets, evaluating on highly variable genes versus all genes. (B) Same as a, but showing percent increase in mean Pearson correlation from ATAC only to DNA+ATAC. Solid diagonal lines indicate y=x.

In order to assess whether this observed generalizability is due to lowly variable (likely housekeeping) genes, we also evaluated the same models using subsets of the held-out test genes that were highly variable (Note S1). As expected, all models had difficulty predicting the expression of highly variable genes, but models using ATAC were more resilient. When considering models trained and tested on the same cell type, the relative increase in mean Pearson correlation from DNA-only to DNA+ATAC was greater when evaluating on highly variable genes compared to all test genes (Fig. 3a). We observed the same trend in the relative performance improvement from ATAC-only to DNA+ATAC (Fig. 3b). Interestingly, we also observed these patterns on the cross-cell type prediction tasks (Figs. S5–S8), although GEx of some cell types seemed to be inherently easier to predict. Taken together, these results suggest that the ATAC-augmented model is less biased by lowly variable genes while being capable of predicting on other cell types.

### 3.3 Positional attribution scores of input channels aligned with chromatin accessibility

To better understand our ATAC-augmented sequence-to-expression model, we examined attribution scores corresponding to each input as determined by the SHAP DeepExplainer (Lundberg and Lee 2017), starting with the ATAC channel. We observed that the ATAC input heavily influenced our model’s output, and that the largest mean ATAC attribution scores aligned with the mean ATAC track peak, slightly upstream of the TSS (Fig. S9, details in Note S3). For highly expressed and accessible genes, ATAC attribution scores were highly variable 500–1000 bp downstream the TSS, indicating that accessibility in this section of the 5′ untranslated region was relevant to model predictions. Thus, our DNA+ATAC model learned TSS-relative position-specific patterns in chromatin accessibility.

**Figure 4:**
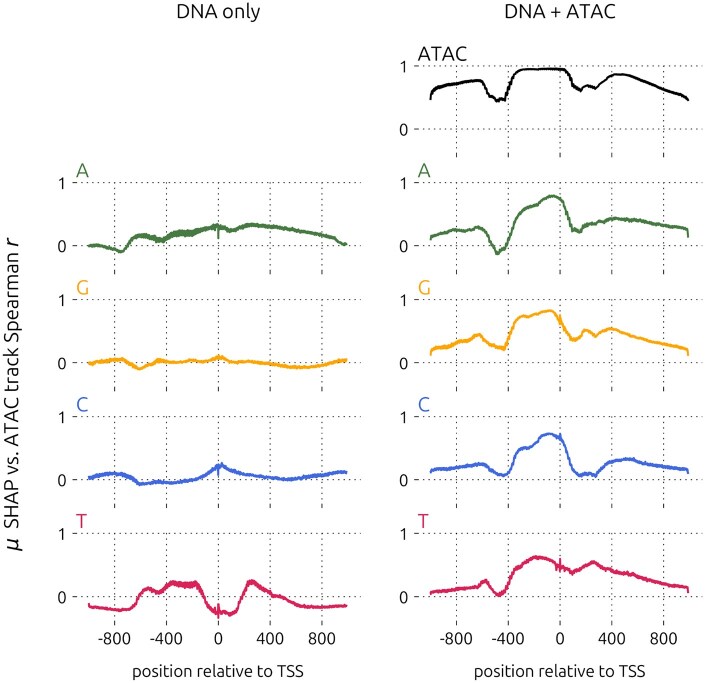
Positional attribution score correlation with accessibility across all input channels of the DNA-only and DNA+ATAC CD4 T cell models. Data shown include all test genes (concatenated from all CV folds).

Next, we compared positional attribution scores in the DNA input channels (Fig. S10). Compared to the baseline model, the variability in the SHAP scores of the DNA channels of the augmented model more closely mirrored variability in the ATAC channel. This was clearly reflected in the Spearman correlation coefficients of mean SHAP scores with the ATAC input track values at each position (Fig. 4). Correlation coefficients were generally close to zero in the DNA-only model, taking on values between −0.461 and 0.372. In the DNA+ATAC model, SHAP scores in the ATAC channel correlated highly with the input ATAC track, with a minimum of 0.224 and maximum of 0.960, and coefficients in the DNA channels followed a similar pattern to those in the ATAC channel, with values between −0.303 and 0.830. Hence, we surmise that the ATAC input feature guided the training of model weights in the DNA channels.

### 3.4 Further improvement with pre-training and replacement of GEx with higher quality data

We questioned whether model performance could be improved by replacing gene expression values with those from a matched more deeply sequenced single-cell dataset. Comparing the gene expression values between multiome and single-cell RNA-seq PBMC datasets, we observed that the single-cell GEx data were less sparse (Fig. S11a and b) and were more highly correlated with auATAC values (Fig. S11c). When we re-trained PBMC models on gene expression data from single-cells, we observed a substantial improvement in the performance of the DNA+ATAC model, as well as the ablated models (Fig. S12). These improvements also extended to highly variable gene expression prediction and generalizability across cell types. Further, we found that model performance was correlated with dataset statistics such as read depth and GEx variance-to-mean ratio across all datasets (details in Note S4).

**Figure 5:**
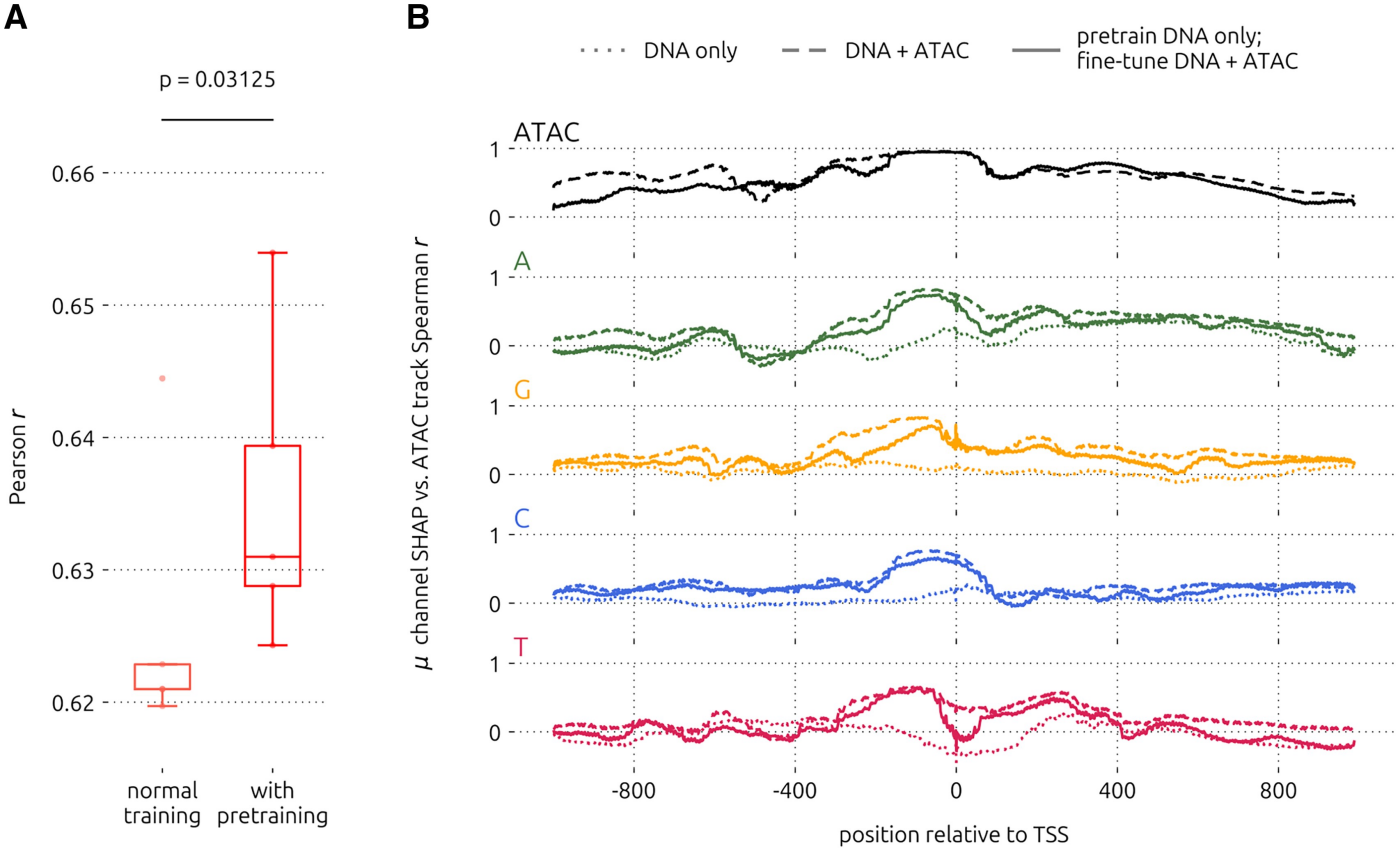
Performance improvement with pre-training. (A) Performance improvement with the pre-training strategy, using single-cell (sc) CD4 T cell GEx data. Boxplots show test results from five-fold CV. Significance was determined by one-sided Wilcoxon signed-rank test. (B) Positional attribution score correlation with the accessibility input channel, for the DNA-only, DNA+ATAC, and pretrained CD4 T cell (sc) models.

To explore an additional strategy that could improve the learning of sequence patterns predictive of gene expression, we tested whether fine-tuning the pre-trained single-cell CD4 T cell DNA-only model with DNA and ATAC would help. After initializing a new DNA+ATAC model with weights copied from a DNA-only model, we trained the augmented model using both sequence and accessibility inputs, as described in Methods. We found that this strategy significantly improved performance on held-out test sets, compared to the DNA+ATAC model trained from randomly initialized weights (Fig. 5a; *P*=0.0313, one-sided Wilcoxon signed-rank test). Moreover, we observed a slight change in the positional correlation of channel attribution scores with the input ATAC tracks (Fig. 5c). With the pre-training strategy, Spearman correlation coefficients were generally similar to or lower than those obtained with the DNA+ATAC model. These observations suggest that this strategy balances information learned from sequence and accessibility. Thus, fine-tuning a pre-trained sequence-only model with both sequence and accessibility is a viable strategy for boosting performance and it may allow the learning of more nuanced sequence patterns as determined by *post hoc* explainers.

### 3.5 Sequence pattern attribution improved with ATAC-seq input

**Figure 6:**
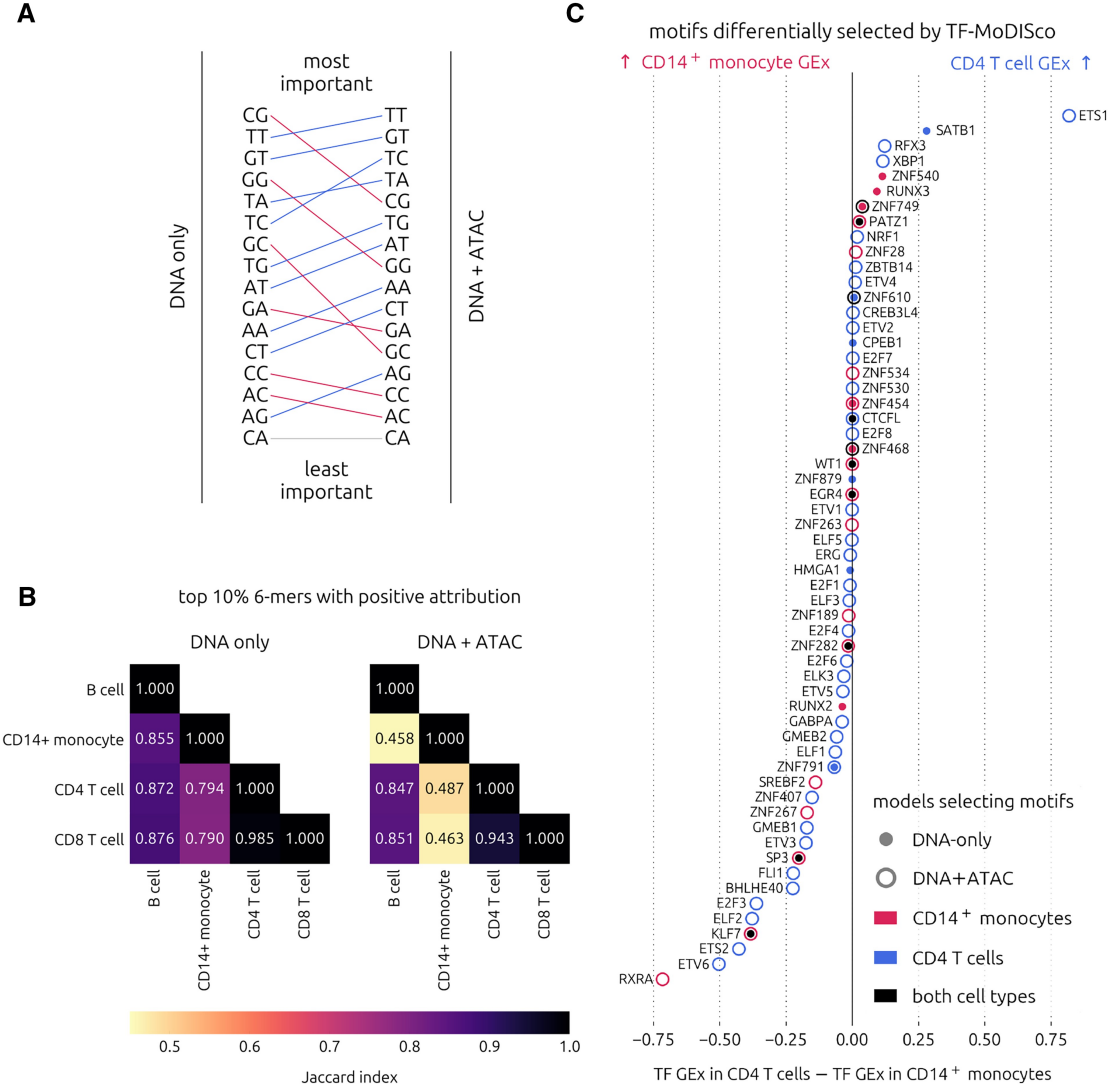
*k*-mer attribution and TF-MoDISco motif comparison between the DNA-only and DNA+ATAC models. (A) Change in rank of mean 2-mer importance from the DNA-only to the DNA+ATAC models, across all folds, random seeds, and PBMC (sc) cell types. (B) Jaccard index of the top 10% of 6-mers with positive attribution between the PBMC cell types, with the right triangle showing similarities within the DNA+ATAC models and the left triangle showing similarities within the DNA-only models. (C) TF-MoDISco hits differentially selected in CD14${\;}^{+}$ monocyte and CD4 T cell models (best Tomtom *q*-value across CV folds < 0.01), ranked by difference in TF GEx.

For an unbiased systematic comparison of sequence pattern attribution scores between our DNA+ATAC and the DNA-only models, we analyzed all instances of *k*-mers in our input sequences. First, we ranked all 2-mers by their mean attribution scores and averaged them across PBMC cell types. We found many differences in the 2-mer importance score rankings between the two model types, the most noteworthy change being that predictions became less reliant on the presence of CpG dinucleotides in the augmented version (Fig. 6a). This finding is a good indicator that allowing the ATAC-seq input to explain some of the variability in gene expression enables the detection of sequence patterns that are less correlated with accessibility. We also observed that reverse complements of dimers were often ranked very differently, indicating that *k*-mer orientation is relevant to gene expression prediction.

Since cell type-specific gene expression is governed by higher-level patterns, we also compared 6-mer importances across cell types. We found that the overlaps between the top 10% of 6-mers among instances with positive attribution in the PBMC cell types approximated the biological similarities of these cell lineages more closely when we used the attribution scores from the DNA+ATAC models (Fig. 6b). The CD4 and CD8 T cell subsets are known to be highly similar while B cells and T cells are both members of the lymphoid lineage, sharing some transcriptional programs. CD14${\;}^{+}$ monocytes are members of the myeloid lineage, and are therefore the most distant from the three other cell types tested here. These relations are best reflected in the pairwise Jaccard indices between the top 6-mers obtained using the augmented models. We observed a similar pattern when repeating this analysis using mean scores across all 6-mer instances, including negative ones (Fig. S13). Altogether, our *k*-mer analyses demonstrate that sequence pattern attribution improved with the addition of ATAC-seq as an input feature.

To verify whether known cell type-specific motifs were detected, we also ran TF-MoDISco (Shrikumar et al. 2020) on SHAP scores of promoters with auATAC greater than the mean for a cell type and matched resulting patterns to known motifs using Tomtom (Gupta et al. 2007). Comparing motifs with positive attribution discovered for CD14${\;}^{+}$ monocytes and CD4 T cells, we found that there were indeed motifs uniquely found in one cell type using the DNA+ATAC models that were not found using the DNA-only models (Fig. 6c). For example, RXRα is known to be a major TF involved in CD14${\;}^{+}$ monocyte maintenance (Núñez et al. 2010) with little evidence of any role in lymphocytes (supported by major difference in GEx, which is 0.75 in CD14${\;}^{+}$ monocytes and 0.03 in CD4 T cells), and its motif (RXRA) was uniquely discovered using the CD14${\;}^{+}$ monocyte DNA+ATAC model. On the other end of the spectrum, Ets1 is known to have a major role in CD4 T cells (Garrett-Sinha 2023) (supported by major difference in GEx, which is 0.05 in CD14${\;}^{+}$ monocytes and 0.86 in CD4 T cells), and its motif (ETS1) was uniquely discovered with the CD4 T cell DNA+ATAC model. Further, the augmented model allowed for the discovery of motifs corresponding to TFs with central roles in both cell types (Fig. S14), such as CREB1 (Wen et al. 2010) (supported by similar GEx values of 0.55–0.57). Interestingly, we also found that the motif corresponding to GATA2, a pioneer TF with a major role in the development of both cell types (master regulator in hematopoietic stem and progenitor cells) but whose expression is downregulated in mature cells (Aktar and Heit 2023) (GEx = 0 in both cell types), was discovered using only the DNA+ATAC models. Similar observations have been made in previous work investigating the relative contributions of sequence and pre-existing chromatin accessibility to TF binding (Srivastava et al. 2021, Arora et al. 2023). Since pioneer TFs can bind inaccessible chromatin, this finding demonstrates that our augmentation strategy allows for the discovery of TFs influencing gene expression independently of chromatin accessibility.

Next, we asked whether our models could predict promoter variant effects using the GTEx eQTL benchmark dataset curated by Jaganathan et al. (2025). For the models using ATAC-seq as input, we supplied the same accessibility track for the alternative sequence as for the reference, as we did not have access to paired RNA-seq and ATAC-seq data for the variants. This was not an ideal setup, as eQTLs are likely to affect accessibility as well as expression. We observed that regardless of the input features used, our models were poor predictors of variant effects (Fig. S15). This is likely due to several reasons, the primary one being that our models predict GEx, defined as a probability value, instead of transcript abundance. Moreover, the length of input sequences was limited to 2 kb, which made it difficult to learn nuanced patterns such as single-nucleotide variant effects. Finally, our models were trained individually on different cell types, and therefore are not expected to generalize well to the heterogeneous biological sources of the benchmark dataset, especially since many eQTLs are cell type-specific (Natri et al. 2024).

### 3.6 Increasing input length did not reduce the benefit imparted by the ATAC-seq input

**Figure 7:**
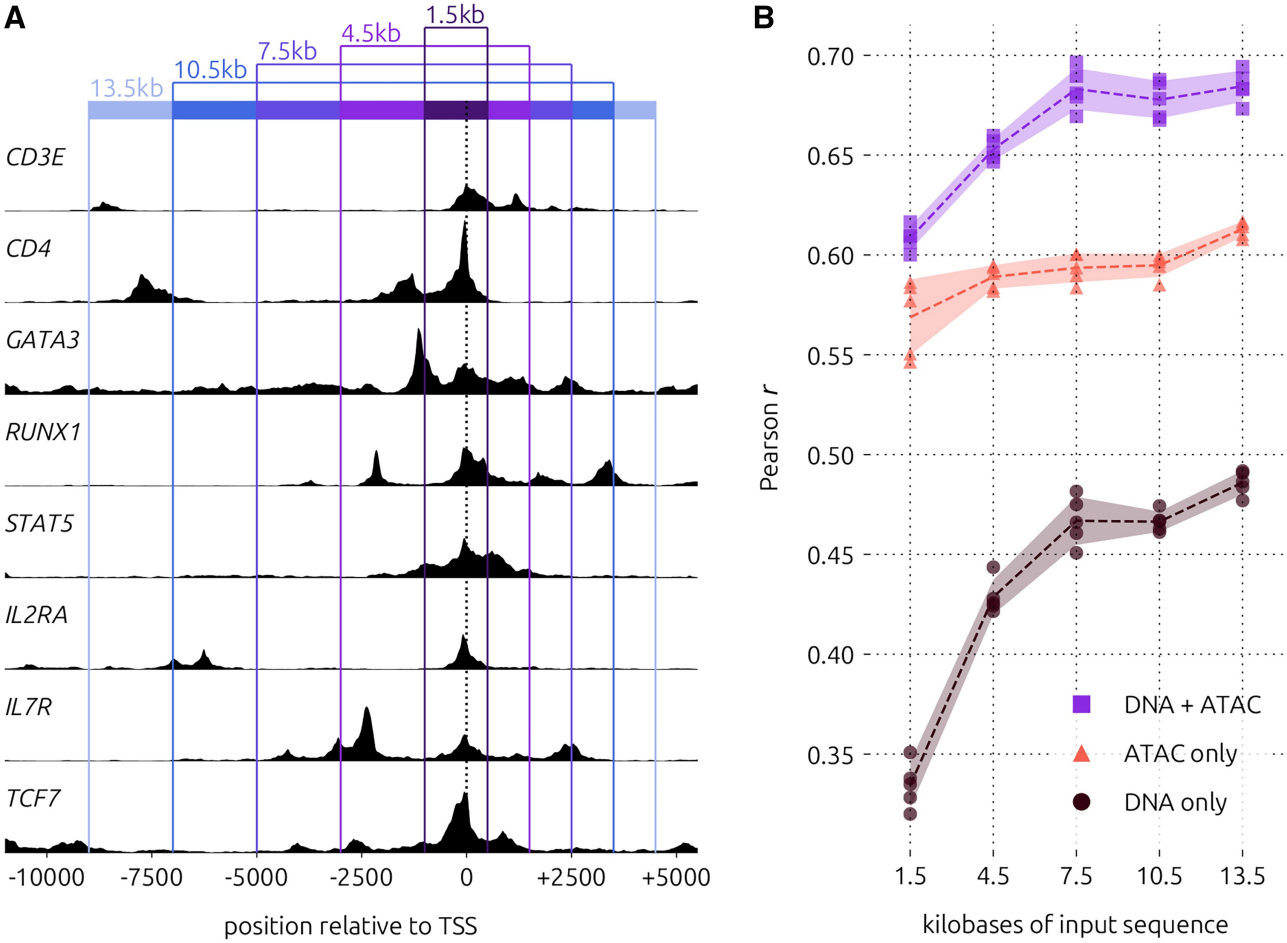
Performance with respect to input length. (A) Normalized chromatin accessibility tracks along promoters of eight representative genes known to play important roles in CD4 T cells, for reference. Input regions used in this experiment are marked relative to the TSS. (B) Pearson correlation coefficient for each of five model replicates trained on CD4 T cells, using the input regions depicted in panel a. Dashed lines indicate means, and shaded regions indicate standard deviations.

To determine the effect of sequence length on model performance with the additional accessibility input feature, we trained new augmented and ablated models to predict GEx in CD4 T cells using five different input lengths ranging from 1.5 to 13.5 kb (Fig. 7a, Table S5, Methods). These lengths were based on those used in previous studies (Agarwal and Shendure 2020, Li et al. 2022) and encompassed regulatory elements including enhancers up to 9 kb upstream or 4.5 kb downstream of the TSS. We found that there was an overall improvement in performance across all model types as the input sequence length was increased, and the DNA-only model improved the most. However, there was still a substantial gap between the DNA-only model (mean Pearson r=0.486), the ATAC-only model (mean Pearson r=0.612), and DNA+ATAC model (mean Pearson r=0.684) using 13.5 kb input sequences. The performance gap between the DNA+ATAC and ATAC-only models grew as the input length increased, but the DNA-only model did not reach nearly the same performance as the ATAC-only model at 13.5 kb. Thus, longer input sequences up to 13.5 kb did not neutralize the benefit of our model augmentation strategy.

## 4 Discussion

Here, we demonstrated that augmenting input DNA sequences with chromatin accessibility refined sequence-to-expression modelling. In all datasets we used, performance consistently and significantly improved not only with respect to the baseline DNA-only model, but also with respect to the ATAC-only model. Moreover, our finding that the relative improvement from both DNA-only and ATAC-only to DNA+ATAC increased for highly variable genes (Fig. 3c) implies that this strategy lessened biases by lowly variable (likely housekeeping) genes. To our knowledge, evaluation of sequence-to-expression prediction on highly variable genes has not been reported. Moreover, we found that the performance of each model type on other cell types within the same dataset was similar to its performance on the same cell type, and these trends were not affected when evaluating on only highly variable genes.

Further, attribution scores in the DNA input channels of the augmented sequence-to-expression model increased at accessible sequence positions when we included ATAC-seq as an input feature. We also demonstrated that adding ATAC-seq as an input feature reduced reliance on CpG content and allowed for the detection of cell type-specific 6-mers and motifs despite looking no further than ±1kb from the TSS. Further, we were able to detect motifs of TFs that bind to DNA independent of accessibility (pioneer factors), a finding in line with those of Srivastava et al. (2021) and Arora et al. (2023). Together, these results suggest that our augmentation strategy may result in improved inference of motif grammar and prediction of single nucleotide variant effects in future studies.

Lastly, we found that fine-tuning of a pre-trained DNA-only model using both DNA and ATAC inputs improved model performance. We speculate that the reason for this improvement is that delaying the use of the strongly predictive accessibility feature might prevent distraction from learning subtle sequence patterns. However, we did not observe major changes in the positional correlation of channel attribution scores with the ATAC input track using the pre-training strategy, compared to normal training. This result implies that applying the fine-tuning approach to existing sequence-to-expression models could yield refined results with minimal effort.

Our study has the following limitations. First, sequencing depth has a major impact on performance, and obtaining deeply sequenced single-cell data is costly. However, the concepts demonstrated in this study can be applied to bulk data as well, provided that both transcriptomic and accessibility data are available for a homogeneous biological sample. In this case, gene expression would be represented as transcript abundance rather than a probability value, which can only be calculated if many replicates exist for the same cell type. This representation is unique to our study and may be beneficial for inference of cis-regulatory grammar, but it is not ideal for variant effect prediction. Another drawback of our study is that 2 kb input sequences provided limited data for model training and excluded distal enhancers, which are known to affect highly variable genes in particular (Bergman et al. 2022). However, cell type-specific sequence patterns were recovered by our augmented model nonetheless, and models trained on 13.5 kb input sequences still benefited significantly from our augmentation strategy. Thus, augmenting an even larger model with ATAC-seq as an additional input feature could yield improvements, although it may benefit less compared to the models trained here.

In conclusion, we added context to a plain sequence-to-expression model by including chromatin accessibility as an input feature, and this prompted the learning of refined sequence patterns. This portable sequence-to-expression augmentation strategy may benefit larger models and result in the inference of more nuanced cell type-specific regulatory grammar.

## Supplementary Material

btag199_Supplementary_Data

## Data Availability

Multiome human PBMC, single-cell human PBMC with ADTs, multiome human brain, and multiome human jejunum datasets were obtained from www.10xgenomics.com/datasets. Dataset identifiers are listed in Table S1.

## Code availability

Implemented code is available on GitHub, at https://github.com/lapohosorsolya/accessible_seq2exp and also on Figshare, at https://doi.org/10.6084/m9.figshare.28836278.
