# Supplementary material for: Refining sequence-to-expression modelling with chromatin accessibility
Source: Bioinformatics. 2026 Apr 26;42(5):btag199. doi: 10.1093/bioinformatics/btag199 (PMC13171176; doi:10.1093/bioinformatics/btag199)
Supplement: btag199_Supplementary_Data [file btag199_supplementary_data.zip › Refining_sequence_to_expression_modelling_with_chromatin_accessibility__v5__supplement.pdf]

Supplement to:
Refining sequence-to-expression modelling with chromatin accessibility

Orsolya Lapohos[1,2,3], Gregory J. Fonseca[4,2*], and Amin Emad[1,2,3,5,6*]

[1]Department of Quantitative Life Sciences, McGill University, Montreal, Canada
[2]Meakins-Christie Laboratories, Research Institute of the McGill University Health Centre, Montreal, Canada
[3]Mila, Quebec AI Institute, Montreal, Canada
[4]Department of Medical Sciences, Khalifa University, Abu Dhabi, United Arab Emirates
[5]Department of Electrical and Computer Engineering, McGill University, Montreal, Canada
[6]The Rosalind and Morris Goodman Cancer Institute, Montreal, Canada

∗ Corresponding authors: amin.emad@mcgill.ca, gregory.fonseca@mcgill.ca

---

Contents

List of Abbreviations **2**

Mathematical Modelling **3**

Supplementary Notes **4**

1 Processing and annotation of multiome data 4
2 Processing and annotation of single-cell data 4
3 ATAC channel attribution scores versus accessibility 5
4 Dataset quality versus model performance 7

Supplementary Tables **9**

Supplementary Figures **16**

References **30**

List of Abbreviations

**ADT**	antibody-derived tag
**ATAC-seq**	assay for transposase-accessible chromatin sequencing
**auATAC**	area under the normalized ATAC-seq track
**auROC**	area under the receiver operating characteristic curve
**bp**	base pair
**CNN**	convolutional neural network
**CpG**	C followed by G
**CV**	cross-validation
**DNA**	deoxyribonucleic acid
**eQTL**	expression quantitative trait loci
**GEx**	proportion of cells expressing a gene, or probability of gene expression
**ISC**	intestinal stem cell
**kb**	kilobase
***k*-mer**	nucleotide sequence of length k
**MSE**	mean squared error
**OPC**	oligodendrocyte precursor cell
**PBMC**	peripheral blood mononuclear cell
**PRO-seq**	precision run-on sequencing
**RNA-seq**	ribonucleic acid sequencing
**SHAP**	Shapley Additive Explanations
**TF**	transcription factor
**TSS**	transcription start site
**UMI**	unique molecular identifiers
**UMAP**	uniform manifold approximation and projection
**VMR**	variance-to-mean ratio

Mathematical Modelling

The sequence-to-expression model architecture explored in this study is fully described in Supplementary Table S4. The two 1D-CNN blocks early in the model rely on the convolution operation, in which each node of a Conv1d layer learns a kernel K such that the output is

$$f(i) = (S * K)(i) = \sum_{n} S(i+n)K(n)$$

where f is the output feature map, S is the input sequence, K is the kernel, and i is the position along the input sequence. In the first CNN layer, there are 128 kernels, each with length 6. Following this layer is a MaxPool1d layer which takes the maximum value within a window of length 8 in order to subsample the output of the previous layer such that the new output is

$$p(i) = \max_{j=0}^{m-1} f(i \cdot s + j)$$

where p is the new output feature map, s is the stride of the pooling window ($s = 8$), and j iterates over the pooling window positions. Another CNN block (Conv1d and MaxPool1d) follows the first block, before flattening and proceeding to the fully-connected feed-forward network (see Supplementary Table S4 for details).

Supplementary Notes

1 Processing and annotation of multiome data

Human single nucleus multiome ATAC and gene expression datasets for peripheral blood mononuclear cells (PBMC), brain, and jejunum were sourced from 10x Genomics (Supplementary Table S1, Data availability). For each multiome dataset, the files with suffixes `filtered_feature_bc_matrix.h5` and `atac_fragments.tsv` were downloaded (see Data availability).

In order to annotate nuclei by cell type, each gene expression matrix was analyzed with the Scanpy (Wolf et al., 2018) package. Briefly, cells with a proportion of mitochondrial unique molecular identifiers (UMIs) greater than 5% of total UMIs were removed, and multiplets were filtered using a total UMI threshold of two standard deviations above the mean. Then, gene expression values were library size-normalized and log-transformed for the identification of highly variable genes using default settings in Scanpy. Nuclei were clustered using the Leiden algorithm (Traag et al., 2019), and ranked differentially expressed genes were obtained between each cluster by Mann-Whitney test. Clusters were annotated manually, by comparing cluster-specific genes with cell type marker genes found by literature search and corroborated with The Human Protein Atlas (Karlsson et al., 2021; Uhlen et al., 2019). Cell type marker genes are indicated in Supplementary Table S2. Four major cell types were used from each dataset. Among PBMCs, we chose B cells, CD14$^+$ monocytes, CD4 T cells, and CD8 T cells. Brain cells were divided into astrocytes/microglia, oligodendrocytes, neurons, and oligodendrocyte precursor cells (OPCs). Cells from the jejunum were divided into epithelial, stromal (immune), stromal (other), and intestinal stem cells (ISCs). Uniform Manifold Approximation and Projection (UMAP) (Mcinnes et al., 2020) graphs of gene expression in each multiome dataset are shown in Supplementary Figure S1A-C, with each major cell type indicated by colour. Cell numbers by cell type are indicated in Supplementary Table S3.

2 Processing and annotation of single-cell data

A human PBMC single-cell gene expression dataset with cell surface marker antibody-derived tags (ADTs) was also obtained from 10x Genomics (Supplementary Table S1, Data availability). ADTs were denoised and scaled by background using the DSB method (Mulè et al., 2022) built into the MUON package (Bredikhin et al., 2022). Cells were then filtered, transformed, and clustered based on gene expression, as described in the previous section. The levels of cell type-specific DSB-normalized ADTs in each cluster were then used to annotate the same four major cell types as in the PBMC multiome dataset (numbers in Supplementary Table S3).

3 ATAC channel attribution scores versus accessibility

Given that ATAC alone is a strong predictor of GEx—considering auATAC as a naïve predictor of GEx, as well as the performance of the ATAC-only model—we wanted to determine its contribution to model outputs for different categories of genes.

Gene binning. We sorted genes into low, intermediate, and high bins, separately by accessibility and gene expression (Supplementary Figure S9A). To find appropriate binning thresholds, we used an automated knee-point detection algorithm for area under the normalized 2kb ATAC input track (auATAC) within the input sequence range and for GEx (probability of gene expression), separately. Briefly, genes were sorted in ascending order, for each feature. Then, the kneed package (Satopa et al., 2011) was used to normalize feature values and obtain a difference curve. The maximum of this difference curve was chosen as the first knee point. To obtain a second knee point, the sorted list of genes was truncated using the first knee point and the knee-point detection process was repeated. The two knee points calculated for each feature (auATAC and GEx) were used to sort genes into low, intermediate, and high bins. Finally, a gene contingency table with 9 categories was obtained by intersecting these bins between auATAC and GEx.

Accessibility attribution analysis. For 97.9% of genes, GEx tended to increase with auATAC in CD4 T cells (Supplementary Figure S9A). A few genes (348) had high auATAC and low GEx, while a smaller number of genes had low auATAC and high GEx (60). We suspected that temporary silencing/pausing or long mRNA half-lives may explain these rare discrepancies.

Discrepancies versus mRNA half-life. We investigated the half-lives of genes in the high GEx $\cap$ low auATAC category by examining their distribution within the overall distribution of a general aggregated mRNA half-life meta-feature obtained from a previous publication (Agarwal and Kelley, 2022) (file: 13059_2022_2811_MOESM3_ESM.xlsx). However, we did not find any concordance between these genes and mRNA half-life (Supplementary Figure SN3.1A).

Discrepancies versus promoter-proximal pausing. To investigate promoter-proximal pausing, we obtained three human PBMC CD4 T cell precision run-on sequencing (PRO-seq) samples published previously (Danko et al., 2018), downloaded from the Gene Expression Omnibus (accession: GSE85337) in the form of pre-processed BigWig files. Since these had been mapped to a different reference genome, corresponding gene coordinates were extracted from the GENCODE GRCh37 backmap reference GTF file (Frankish et al., 2022). Then, the three samples were aggregated into one before calculating pausing indices. The pausing index for each gene was calculated by dividing the number of reads within the first 1kb downstream the TSS by the number of reads along the rest of the gene body (on the coding strand, and only for genes at least 3kb in length) as described elsewhere (Adelman and Lis, 2012). Analyzing the obtained pausing indices for each gene, we observed that in the low GEx $\cap$ high auATAC category, only 25.2% of genes had a pausing index greater than 2 (Supplementary Figure SN3.1B). Altogether, these observations suggest that neither mRNA half-life nor

promoter-proximal pausing can explain all discrepancies between GEx and auATAC—these rare occurrences may be better explained by technical limitations.

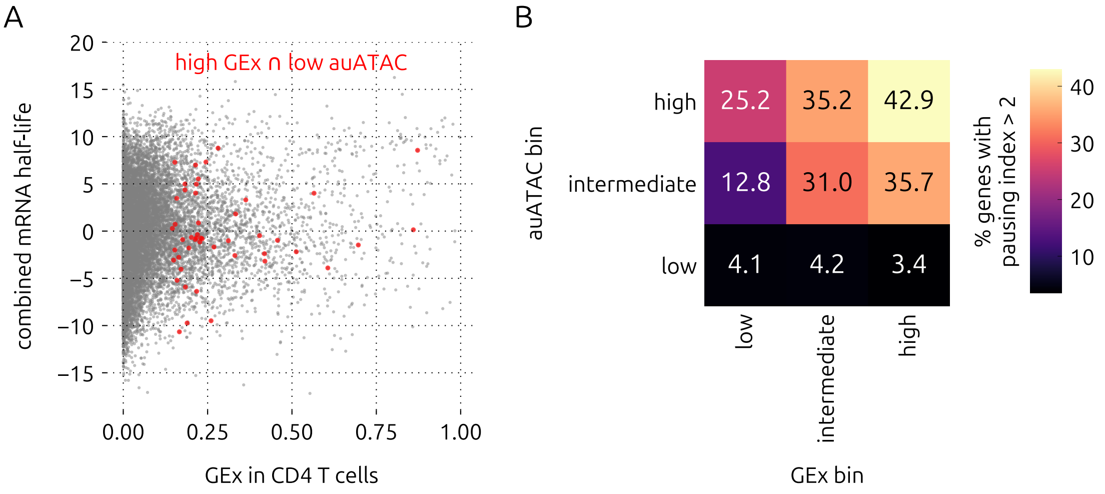

Supplementary Figure SN3.1: mRNA half-life and proximal pausing analysis in CD4 T cells. A. Combined general mRNA half-life "meta-feature" (Agarwal and Kelley, 2022) versus GEx in CD4 T cells from the multiome PBMC dataset. Genes in the high GEx and low auATAC category are coloured red. B. The percentage of genes in each CD4 T cell category with a pausing index greater than 2, as determined from PRO-seq data of human PBMC CD4 T cells (Danko et al., 2018).

Positional attribution scores for accessibility. We computed positional attribution scores in the ATAC channel and averaged them for each gene category (Supplementary Figure S9B). We observed that the ATAC input heavily influenced our model's output, and that the largest mean ATAC attribution scores aligned with the mean ATAC track peak, slightly upstream of the TSS. Interestingly, relatively large values in the ATAC input track (∼0.25) did not contribute positively to the model's output 400-450bp upstream of the TSS and 100-400bp downstream of the TSS. For genes with high auATAC, ATAC attribution scores were highly variable 500-1000bp downstream the TSS, indicating that accessibility in this section of the 5' untranslated region was deemed relevant to the model. Overall, these results show that our DNA+ATAC model learned TSS-relative position-specific patterns in chromatin accessibility.

4 Dataset quality versus model performance

After having trained sequence-to-expression models on several datasets, we suspected that differences in underlying statistics may have affected model performance. We investigated whether the number of reads per cell (Supplementary Figure SN3.2), the number of pooled cells (Supplementary Table S3), or the variation in GEx values impacted model training. We observed that performance increased linearly with mean log UMIs per pooled cell (Supplementary Figure SN3.3, top). This trend occurred in the DNA-only, ATAC-only, and DNA+ATAC models, with R^2 values of 0.49, 0.79, and 0.82, respectively (Supplementary Figure S15, top). The number of pooled cells (Supplementary Figure SN3.3, bottom left) did not have an effect on performance. Finally, we found a trend between mean Pearson correlation and the variance-to-mean ratio (VMR) of GEx values for the ATAC-only and DNA+ATAC models ($R^2 = 0.68$ and 0.53, respectively), but not the DNA-only model (Supplementary Figure SN3.3, bottom right). This result further corroborates our finding that including accessibility as an input feature helps to explain some of the variability in gene expression. Altogether, these results demonstrate the importance of sequencing depth in sequence-to-expression modelling.

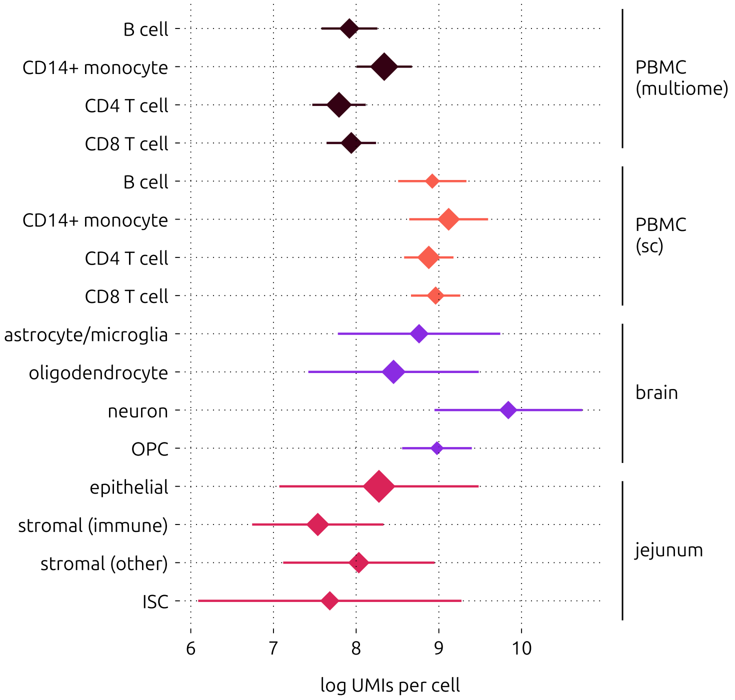

Supplementary Figure SN3.2: Mean log UMIs per cell by cell type in each dataset used in this study. Marker area is proportional to the number of cells pooled for that cell type. Error bars represent standard deviation.

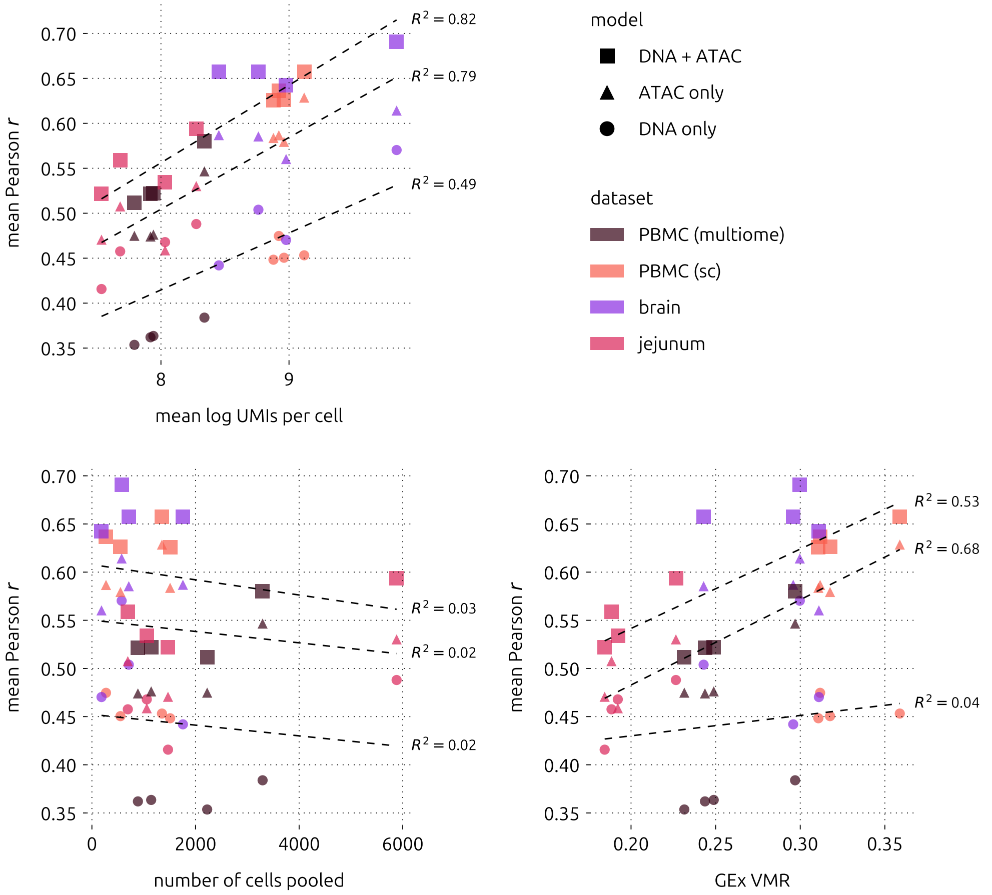

Supplementary Figure SN3.3: Relationship between dataset statistics and model performance. Top: mean Pearson correlation versus mean log UMIs per cell. Bottom left: mean Pearson correlation versus number of cells pooled. Bottom right: mean Pearson correlation versus variance-to-mean ratio (VMR) of GEx in that dataset. Datasets are colour-coded. Marker shapes correspond to model types: DNA+ATAC (square), ATAC only (triangle), and DNA only (circle). For each model type, the line of best fit and its coefficient of determination (R^2) is shown.

Supplementary Tables

S1 Dataset sequencing depth and pre-processing statistics 10

S2 Cell type marker genes used to annotate major cell types 11

S3 Major cell types identified and number pooled in each dataset 12

S4 Architectural details 13

S5 Input lengths used to determine the effect of sequence length on performance 14

S6 Ablation experiment results for all multiome datasets 15

Supplementary Table S1: Sequencing depth and pre-processing statistics of datasets used in this study. ATAC: assay for transposase-accessible chromatin. GEX: gene expression. PBMC: peripheral blood mononuclear cells.

Alias	10x Genomics dataset	10x Genomics pipeline version	Estimated cell number	ATAC mean raw read pairs per cell	ATAC median HQ fragments per cell	GEX mean raw read pairs per cell	GEX median UMI counts per cell	Year published
PBMC (multiome)	PBMC from a Healthy Donor - No Cell Sorting (10k)	v2.0.0	12,012	38,103	11,769	69,129	3,300	2021
PBMC (sc)	5k Human PBMCs Stained with TotalSeqTM-B Human TBNK Cocktail, Chromium NextGEM Single Cell 3'	v8.0.0	5,078	NA	NA	71,818	7,612	2024
brain	Flash-Frozen Human Healthy Brain Tissue (3k)	v2.0.0	3,233	95,897	22,881	122,335	6,966	2021
jejunum	Human Jejunum Nuclei Isolated with Chromium Nuclei Isolation Kit, SaltyEZ Protocol, and 10x Complex Tissue DP (CT Sorted and CT Unsorted)	v2.0.2	10,640	58,571	8,340	17,307	3,676	2023

Supplementary Table S2: Cell type marker genes used to annotate major cell types in multiome datasets. PBMC: peripheral blood mononuclear cells. OPC: oligodendrocyte precursor cell. ISC: intestinal stem cell.

Tissue	Major cell type	Relevant marker genes
PBMC	B cell	*CD19, MS4A1*
	$CD14^+$ monocyte	*CD36, CSF3R, S100A9, SLC8A1, VCAN*
	CD4 T cell	*CD4, IL7R, INPP4B, LEF1*
	CD8 T cell	*CD8A, IL7R, LEF1, NELL2*
brain	astrocyte/microglia	*AQP4, CD83, MERTK, P2RY12*
	oligodendrocyte	*PLP1*
	neuron	*CNR1, GAD2, SNAP25, SYNPR*
	OPC	*PDGFRA*
jejunum	epithelial	*BEST4, DEFA5, PTPRN2, SLC15A1, SLC12A2*
	stromal (immune)	*SEMA4D, MERTK*
	stromal (other)	*CDH19, LRMDA, MYH11, RSPO3, SYT1*
	ISC	*LRMDA, MET*

Supplementary Table S3: Major cell types identified and number pooled in each dataset. PBMC: peripheral blood mononuclear cells. OPC: oligodendrocyte precursor cell. ISC: intestinal stem cell.

Dataset	Identified major cell type	Cell number
PBMC (multiome)	B cell	888
	$CD14^+$ monocyte	3295
	CD4 T cell	2225
	CD8 T cell	1144
PBMC (single cell)	B cell	272
	$CD14^+$ monocyte	1352
	CD4 T cell	1510
	CD8 T cell	551
brain	astrocyte/microglia	712
	oligodendrocyte	1757
	neuron	574
	OPC	185
jejunum	epithelial	5882
	stromal (immune)	1469
	stromal (other)	1059
	ISC	693

Supplementary Table S4: Architectural details. Layer types correspond to `torch.nn` module names. In the input layer, there are 1, 4, or 5 channels, depending on whether accessibility, sequence, or both are included. p represents the probability of nodes to be zeroed out, C_{in} represents the number of input channels, C_{out} represents the number of output channels (filters), k represents kernel size, s represents stride, and d represents dilation.

Block	Layer Type	Parameters	Output Shape
	Input		$x \times 2000 \mid x \in \{1, 4, 5\}$
	Dropout	$p = 0.5$	
1	Conv1d	$C_{\text{in}} = 5$, $C_{\text{out}} = 128$, $k = 6$, $s = 1$, $d = 1$	128×1996
	MaxPool1d	$k = 8$, $s = 8$, $d = 1$	128×249
2	Conv1d	$C_{\text{in}} = 128$, $C_{\text{out}} = 64$, $k = 9$, $s = 1$, $d = 2$	64×233
	MaxPool1d	$k = 8$, $s = 8$, $d = 1$	64×29
	Flatten		1856
	Dropout	$p = 0.5$	
3	Linear		1856
	ReLU		
	Dropout	$p = 0.5$	
	Linear		64
	ReLU		
	Dropout	$p = 0.5$	
	Linear		1

Supplementary Table S5: Input lengths used to determine the effect of sequence length on performance, and corresponding minibatch sizes used during training. All other hyper-parameters remained unchanged from those used in the study, aside from the widths of the convolutional blocks, which had to accommodate the longer input sequences. TSS: transcription start site.

Input Length	Distance Upstream TSS	Distance Downstream TSS	Minibatch Size
1.5 kb	1 kb	0.5 kb	512
4.5 kb	3 kb	1.5 kb	512
7.5 kb	5 kb	2.5 kb	256
10.5 kb	7 kb	3.5 kb	256
13.5 kb	9 kb	4.5 kb	128

Supplementary Table S6: Ablation experiment results for all multiome datasets. Performance metrics on the held-out test sets are shown. MSE: mean squared error. All comparisons within cell type are significantly different by one-sided Wilcoxon signed rank test ($p < 0.05$), except ATAC only versus DNA+ATAC Spearman r for CD4 T cell.

**Dataset**	**Cell type**	**Model**	**Pearson r**	**Spearman r**	**MSE**	R^2
PBMC	B cell	DNA only	0.3620 ± 0.0196	0.5344 ± 0.0063	0.0151 ± 0.0006	0.1162 ± 0.0094
		ATAC only	0.4741 ± 0.0160	0.7042 ± 0.0058	0.0138 ± 0.0007	0.1927 ± 0.0120
		**DNA+ATAC**	**0.5215 ± 0.0163**	**0.7200 ± 0.0059**	**0.0126 ± 0.0005**	**0.2649 ± 0.0154**
	$CD14^+$ monocyte	DNA only	0.3839 ± 0.0133	0.5237 ± 0.0106	0.0247 ± 0.0018	0.1364 ± 0.0062
		ATAC only	0.5466 ± 0.0094	0.7338 ± 0.0079	0.0207 ± 0.0017	0.2770 ± 0.0105
		**DNA+ATAC**	**0.5801 ± 0.0096**	**0.7422 ± 0.0080**	**0.0193 ± 0.0015**	**0.3259 ± 0.0117**
	CD4 T cell	DNA only	0.3536 ± 0.0170	0.5304 ± 0.0084	0.0137 ± 0.0007	0.1037 ± 0.0069
		ATAC only	0.4749 ± 0.0084	0.7127 ± 0.0079	0.0123 ± 0.0006	0.1958 ± 0.0027
		**DNA+ATAC**	**0.5116 ± 0.0090**	**0.7169 ± 0.0052**	**0.0114 ± 0.0005**	**0.2543 ± 0.0085**
	CD8 T cell	DNA only	0.3635 ± 0.0191	0.5391 ± 0.0084	0.0160 ± 0.0010	0.1124 ± 0.0077
		ATAC only	0.4762 ± 0.0101	0.7074 ± 0.0064	0.0145 ± 0.0009	0.1931 ± 0.0043
		**DNA+ATAC**	**0.5222 ± 0.0119**	**0.7171 ± 0.0053**	**0.0132 ± 0.0007**	**0.2657 ± 0.0108**
brain	astrocyte/microglia	DNA only	0.5039 ± 0.0091	0.5933 ± 0.0079	0.0273 ± 0.0011	0.2241 ± 0.0104
		ATAC only	0.5854 ± 0.0045	0.7096 ± 0.0031	0.0243 ± 0.0013	0.3086 ± 0.0089
		**DNA+ATAC**	**0.6574 ± 0.0077**	**0.7506 ± 0.0074**	**0.0203 ± 0.0010**	**0.4211 ± 0.0069**
	oligodendrocyte	DNA only	0.4420 ± 0.0026	0.5895 ± 0.0071	0.0249 ± 0.0012	0.1722 ± 0.0079
		ATAC only	0.5869 ± 0.0163	0.7432 ± 0.0046	0.0208 ± 0.0014	0.3087 ± 0.0174
		**DNA+ATAC**	**0.6575 ± 0.0141**	**0.7679 ± 0.0068**	**0.0173 ± 0.0013**	**0.4248 ± 0.0170**
	neuron	DNA only	0.5703 ± 0.0097	0.6251 ± 0.0089	0.0529 ± 0.0006	0.2980 ± 0.0037
		ATAC only	0.6142 ± 0.0060	0.6966 ± 0.0040	0.0520 ± 0.0013	0.3106 ± 0.0106
		**DNA+ATAC**	**0.6909 ± 0.0045**	**0.7520 ± 0.0039**	**0.0401 ± 0.0007**	**0.4683 ± 0.0056**
	OPC	DNA only	0.4703 ± 0.0153	0.5802 ± 0.0123	0.0361 ± 0.0005	0.1923 ± 0.0098
		ATAC only	0.5603 ± 0.0103	0.7168 ± 0.0043	0.0320 ± 0.0009	0.2846 ± 0.0100
		**DNA+ATAC**	**0.6426 ± 0.0109**	**0.7612 ± 0.0019**	**0.0267 ± 0.0009**	**0.4033 ± 0.0120**
jejunum	epithelial	DNA only	0.4880 ± 0.0113	0.5761 ± 0.0102	0.0212 ± 0.0009	0.2239 ± 0.0094
		ATAC only	0.5302 ± 0.0126	0.6798 ± 0.0055	0.0208 ± 0.0012	0.2369 ± 0.0104
		**DNA+ATAC**	**0.5939 ± 0.0105**	**0.7156 ± 0.0058**	**0.0180 ± 0.0007**	**0.3406 ± 0.0108**
	stromal (immune)	DNA only	0.4157 ± 0.0153	0.5444 ± 0.0139	0.0100 ± 0.0006	0.1533 ± 0.0102
		ATAC only	0.4706 ± 0.0073	0.6592 ± 0.0035	0.0095 ± 0.0007	0.1948 ± 0.0061
		**DNA+ATAC**	**0.5219 ± 0.0107**	**0.6895 ± 0.0053**	**0.0087 ± 0.0006**	**0.2624 ± 0.0079**
	stromal (other)	DNA only	0.4679 ± 0.0131	0.5575 ± 0.0125	0.0136 ± 0.0005	0.1866 ± 0.0134
		ATAC only	0.4587 ± 0.0041	0.6241 ± 0.0045	0.0137 ± 0.0008	0.1807 ± 0.0067
		**DNA+ATAC**	**0.5342 ± 0.0074**	**0.6671 ± 0.0065**	**0.0121 ± 0.0006**	**0.2746 ± 0.0083**
	ISC	DNA only	0.4576 ± 0.0236	0.5701 ± 0.0150	0.0145 ± 0.0005	0.1922 ± 0.0193
		ATAC only	0.5076 ± 0.0092	0.6604 ± 0.0053	0.0139 ± 0.0007	0.2273 ± 0.0116
		**DNA+ATAC**	**0.5589 ± 0.0158**	**0.6979 ± 0.0092**	**0.0125 ± 0.0005**	**0.3045 ± 0.0143**

Supplementary Figures

SN3.1 mRNA half-life and proximal pausing analysis in CD4 T cells 6

SN3.2 Mean log UMIs per cell by cell type in each dataset used in this study 7

SN3.3 Relationship between dataset statistics and model performance 8

S1 10x Genomics multiome datasets 17

S2 Model architecture 18

S3 Naïve predictor performance on the PBMC dataset 18

S4 Cross-cell type performance summary 19

S5 Cross-cell type performance within the PBMC dataset 20

S6 Cross-cell type performance within the brain dataset 21

S7 Cross-cell type performance within the jejunum dataset 22

S8 Cross-cell type performance summary, evaluated on highly variable genes . . 23

S9 Chromatin accessibility and corresponding attribution scores by gene category 23

S10 Positional attribution score distribution across all input channels 24

S11 Comparison of PBMC GEx between multiome and single-cell datasets 25

S12 Cross-cell type performance within the PBMC single-cell dataset 26

S13 Comparison of top 6-mers by mean attribution in the PBMC (sc) dataset . . 27

S14 Motifs discovered by TF-MoDISco for $CD14^+$ monocytes and CD4 T cells . . 28

S15 Promoter variant effect prediction 29

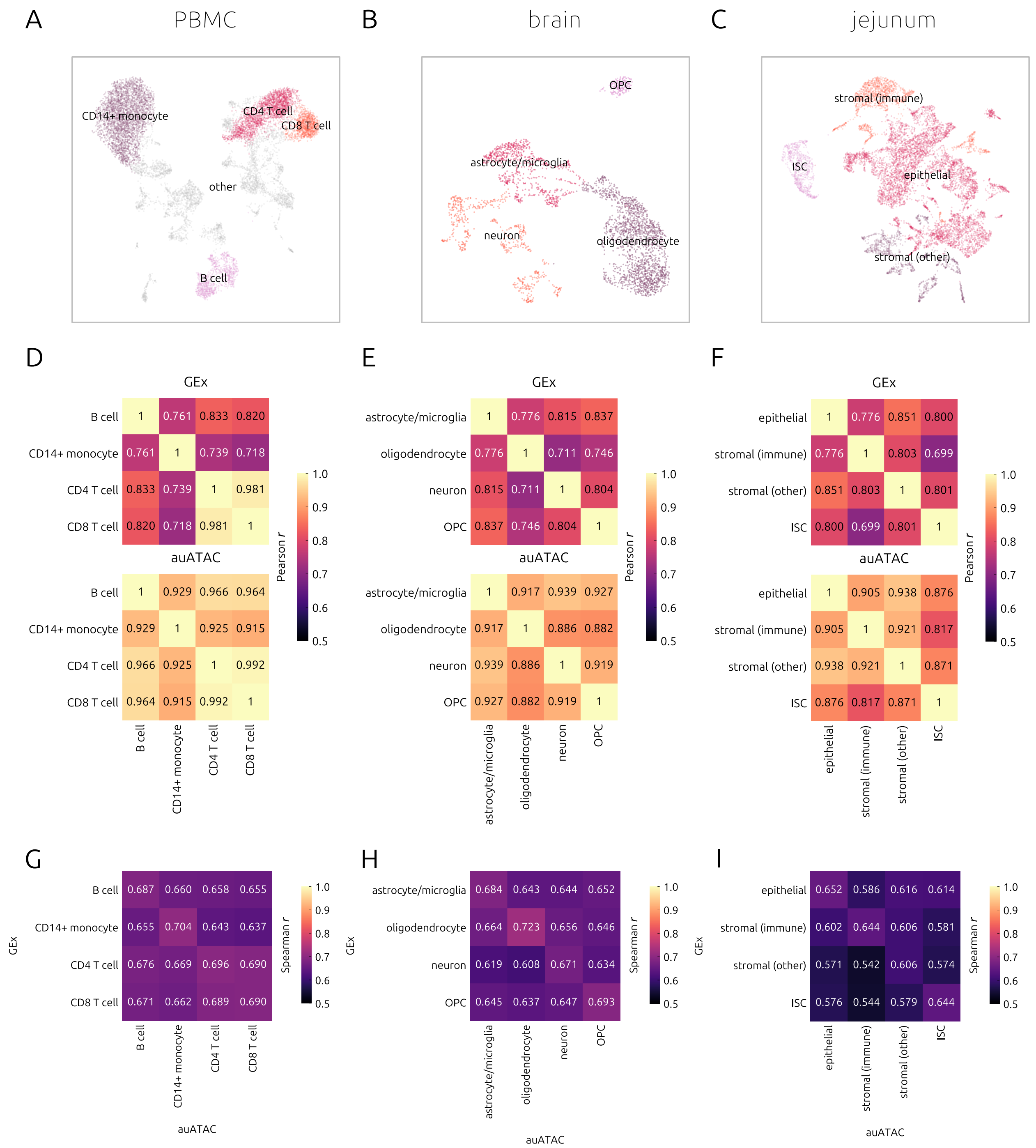

Supplementary Figure S1: 10x Genomics multiome datasets: PBMC (left), brain (center), and jejunum (right). A-C. Uniform manifold approximation and projection graphs of gene expression in 10x Genomics multiome datasets. The 4 major cell types used in each dataset are indicated in colour. D-F. Pearson correlation of GEx (top) and auATAC (bottom) between each major cell type in each multiome dataset. G-I. Spearman correlation between GEx and auATAC in each multiome dataset, within and between each major cell type.

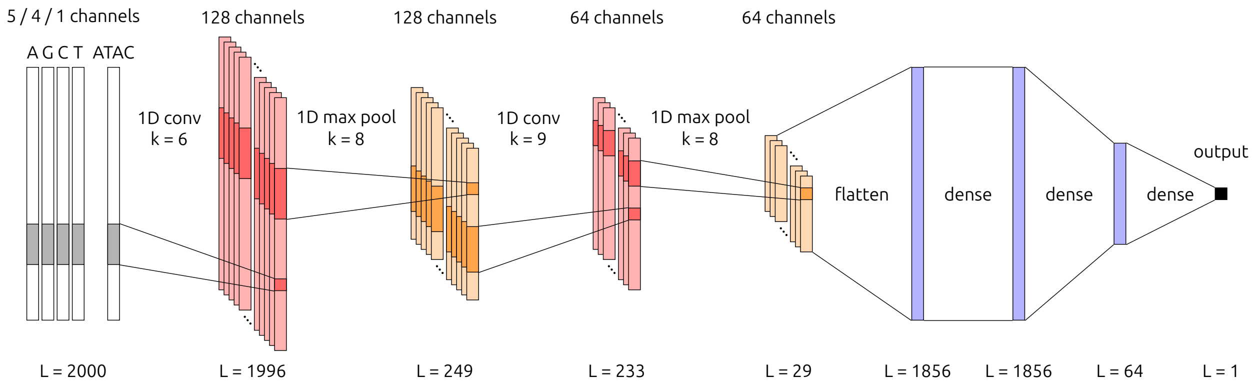

Supplementary Figure S2: Model architecture, adapted from Xpresso (Agarwal et al. 2020). Input channels may consist of 5, 4, or 1 channels, depending on the selected combination of DNA sequence and/or ATAC-seq track inputs. The first part of the model consists of 2 convolutional blocks in which each 1D convolution layer is followed by a 1D max pooling layer. Channel numbers and kernel sizes (k) are indicated for each layer. The second part of the model consists of 3 dense layers with ReLU activation, yielding a single output value.

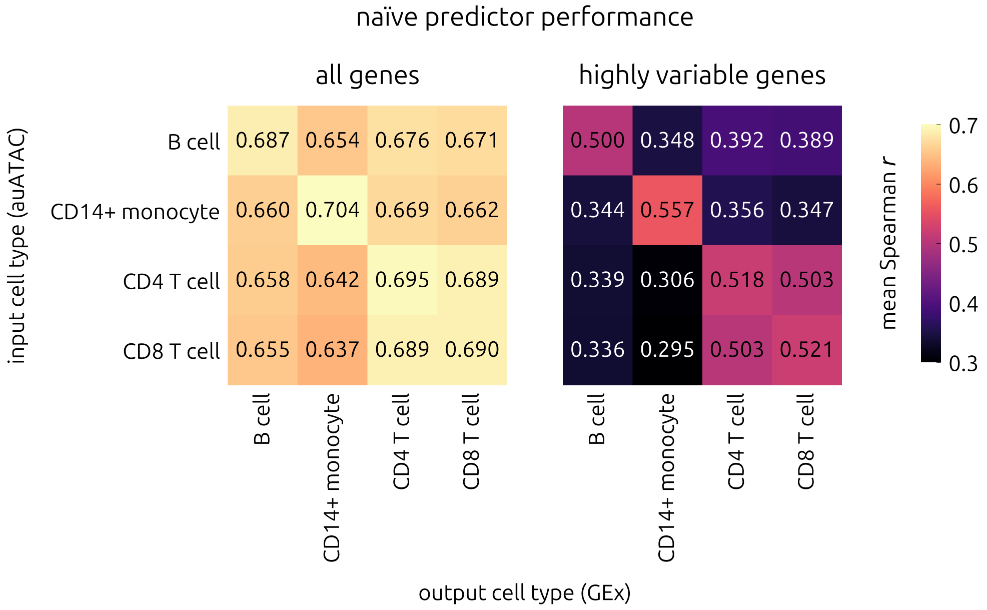

Supplementary Figure S3: Naïve predictor performance on the PBMC dataset. For each fold of 5-fold CV, the naïve predictor outputs the area under the input ATAC input track as a prediction for GEx. Mean Spearman correlation coefficients are shown for each combination of input and output cell type. On the left, evaluation results are shown using all genes in the test sets. On the right side, evaluation was restricted to only highly variable genes in the test sets.

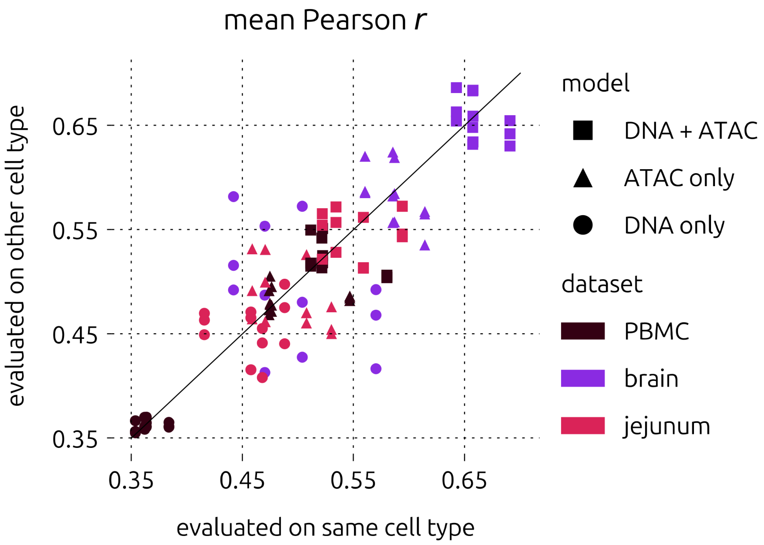

Supplementary Figure S4: Cross-cell type performance summary. Mean Pearson r of models evaluated on held-out test sequences using another cell type from the same dataset (tissue) versus on the cell type used for training. Datasets are colour-coded. Marker shapes correspond to model types: DNA+ATAC (square), ATAC only (triangle), and DNA only (circle).

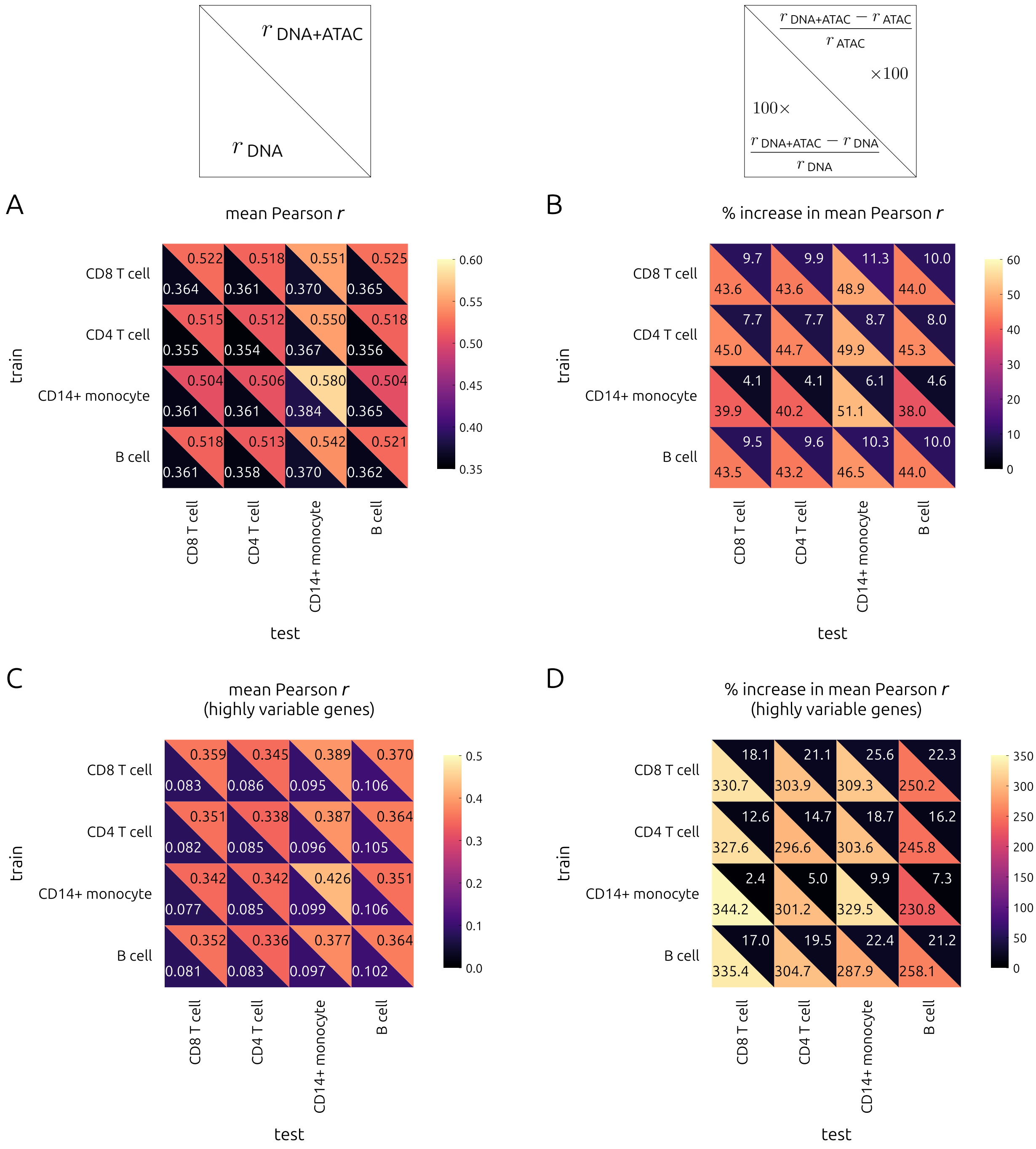

Supplementary Figure S5: Cross-cell type performance within the PBMC dataset. A. Mean Pearson r of models trained on one cell type (rows) and tested on another (columns) for the PBMC dataset. Metrics for the DNA+ATAC model are shown in the top right triangle of each grid square. Metrics for the DNA-only model are shown in the bottom left triangle of each grid square. B. Same as A, but showing mean percent increase in Pearson r of the DNA+ATAC model relative to DNA only (bottom left) and ATAC only (top right). C. Same as A, but evaluated on highly variable genes. D. Same as B, but evaluated on highly variable genes.

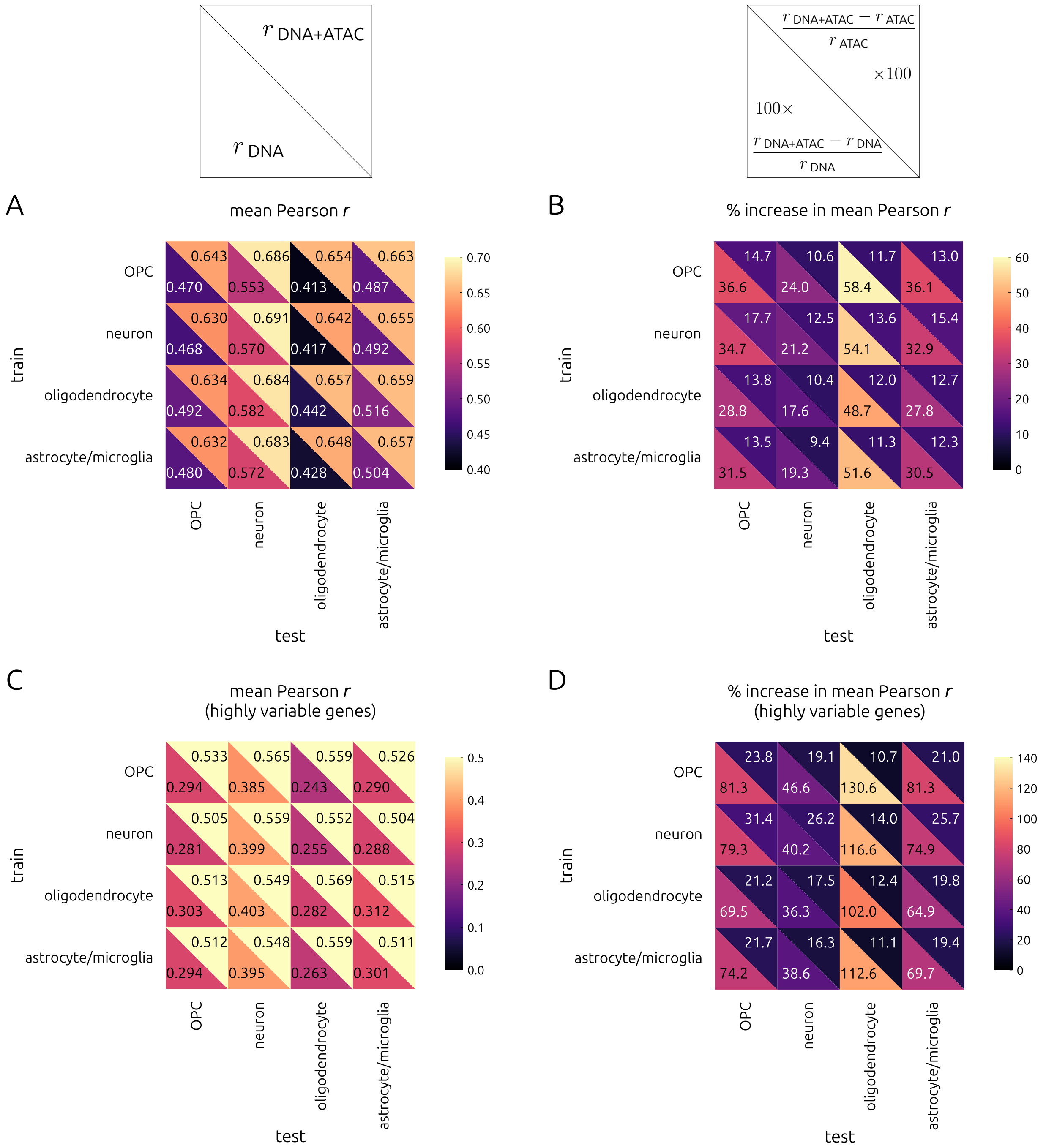

Supplementary Figure S6: Cross-cell type performance within the brain dataset. A. Mean Pearson r of models trained on one cell type (rows) and tested on another (columns) for the brain dataset. Metrics for the DNA+ATAC model are shown in the top right triangle of each grid square. Metrics for the DNA-only model are shown in the bottom left triangle of each grid square. B. Same as A, but showing mean percent increase in Pearson r of the DNA+ATAC model relative to DNA only (bottom left) and ATAC only (top right). C. Same as A, but evaluated on highly variable genes. D. Same as B, but evaluated on highly variable genes.

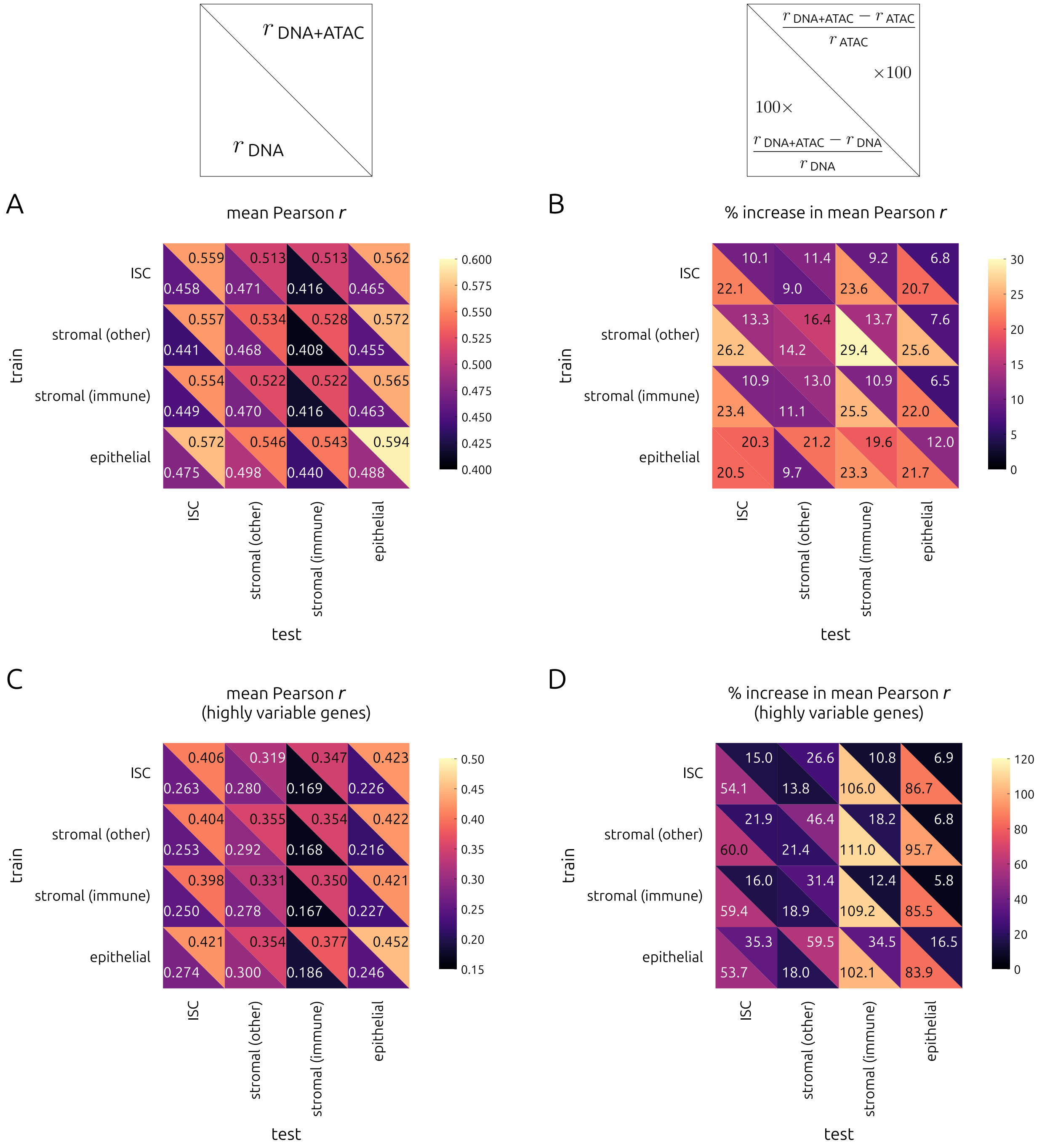

Supplementary Figure S7: Cross-cell type performance within the jejunum dataset. A. Mean Pearson r of models trained on one cell type (rows) and tested on another (columns) for the jejunum dataset. Metrics for the DNA+ATAC model are shown in the top right triangle of each grid square. Metrics for the DNA-only model are shown in the bottom left triangle of each grid square. B. Same as A, but showing mean percent increase in Pearson r of the DNA+ATAC model relative to DNA only (bottom left) and ATAC only (top right). C. Same as A, but evaluated on highly variable genes. D. Same as B, but evaluated on highly variable genes.

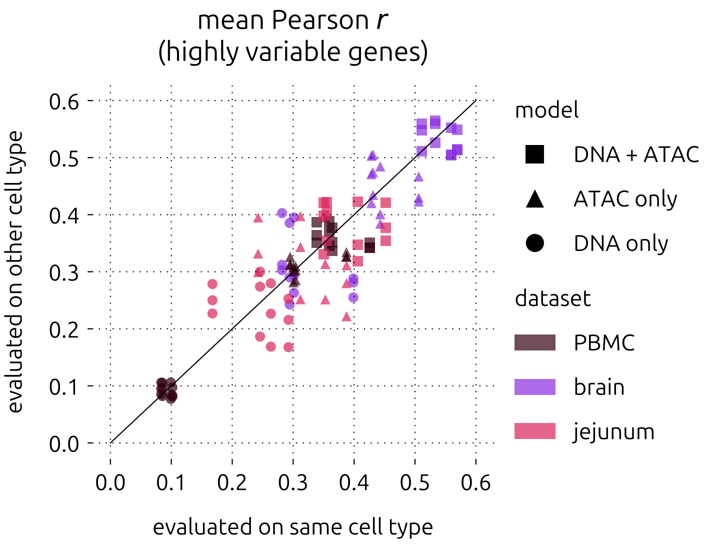

Supplementary Figure S8: Cross-cell type performance summary, evaluated on highly variable genes. Mean Pearson r of models evaluated on held-out test sequences using another cell type from the same dataset (tissue) versus on the cell type used for training. Datasets are colour-coded. Marker shapes correspond to model types: DNA+ATAC (square), ATAC only (triangle), and DNA only (circle).

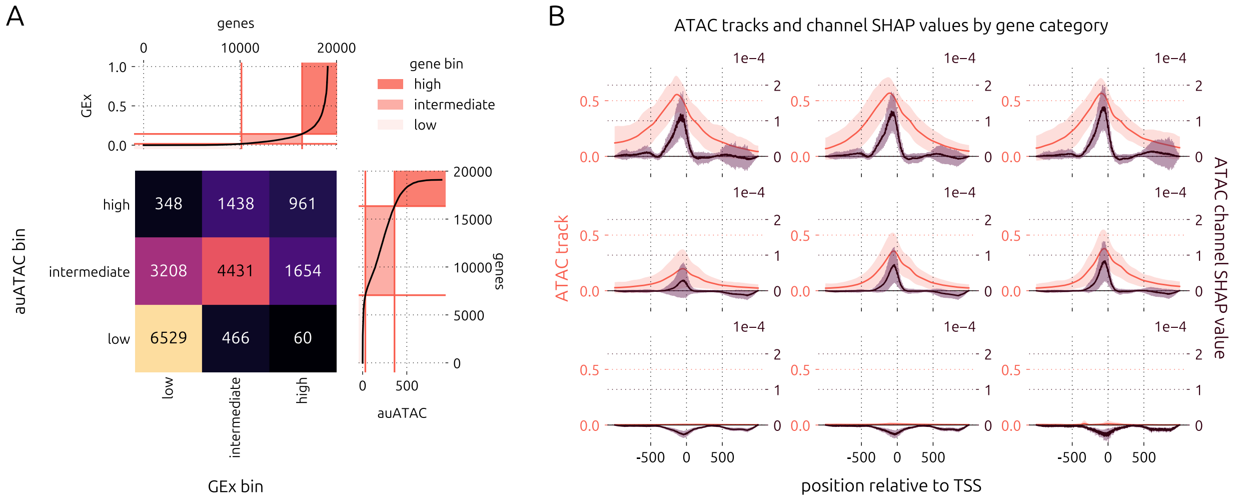

Supplementary Figure S9: Chromatin accessibility and corresponding attribution scores in CD4 T cells, broken down by gene category. A. Gene contingency table generated by double knee point thresholding on GEx and area under ATAC (auATAC) for CD4 T cells. B. ATAC SHAP scores and ATAC input tracks for each category of the gene contingency table shown in A (DNA+ATAC model). Solid lines show means and shaded regions show standard deviations across all genes (after taking mean positional SHAP values across all random seeds).

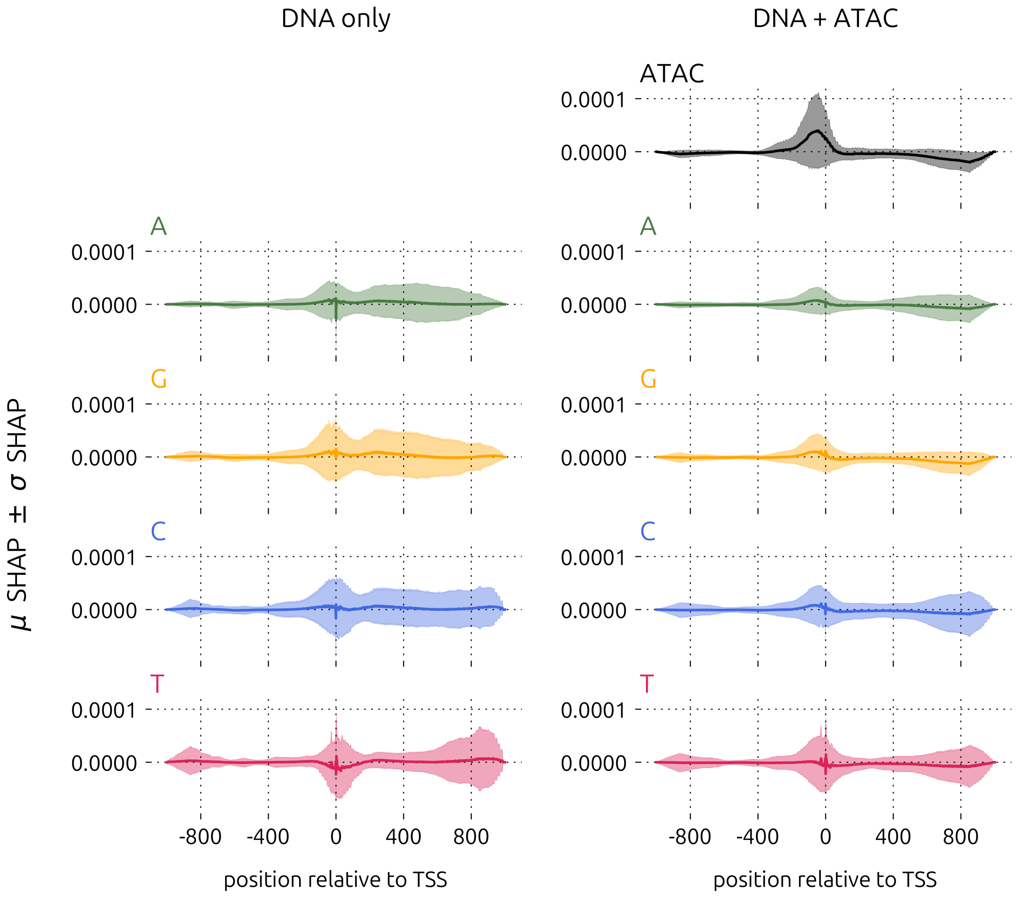

Supplementary Figure S10: Positional attribution score distribution across all input channels of the DNA-only and DNA+ATAC CD4 T cell models. μ and σ SHAP represent the mean and standard deviation of the positional SHAP scores in the indicated channel across 5 random seeds for all test genes (concatenated from all CV folds). Solid lines show μ and shaded regions indicate σ.

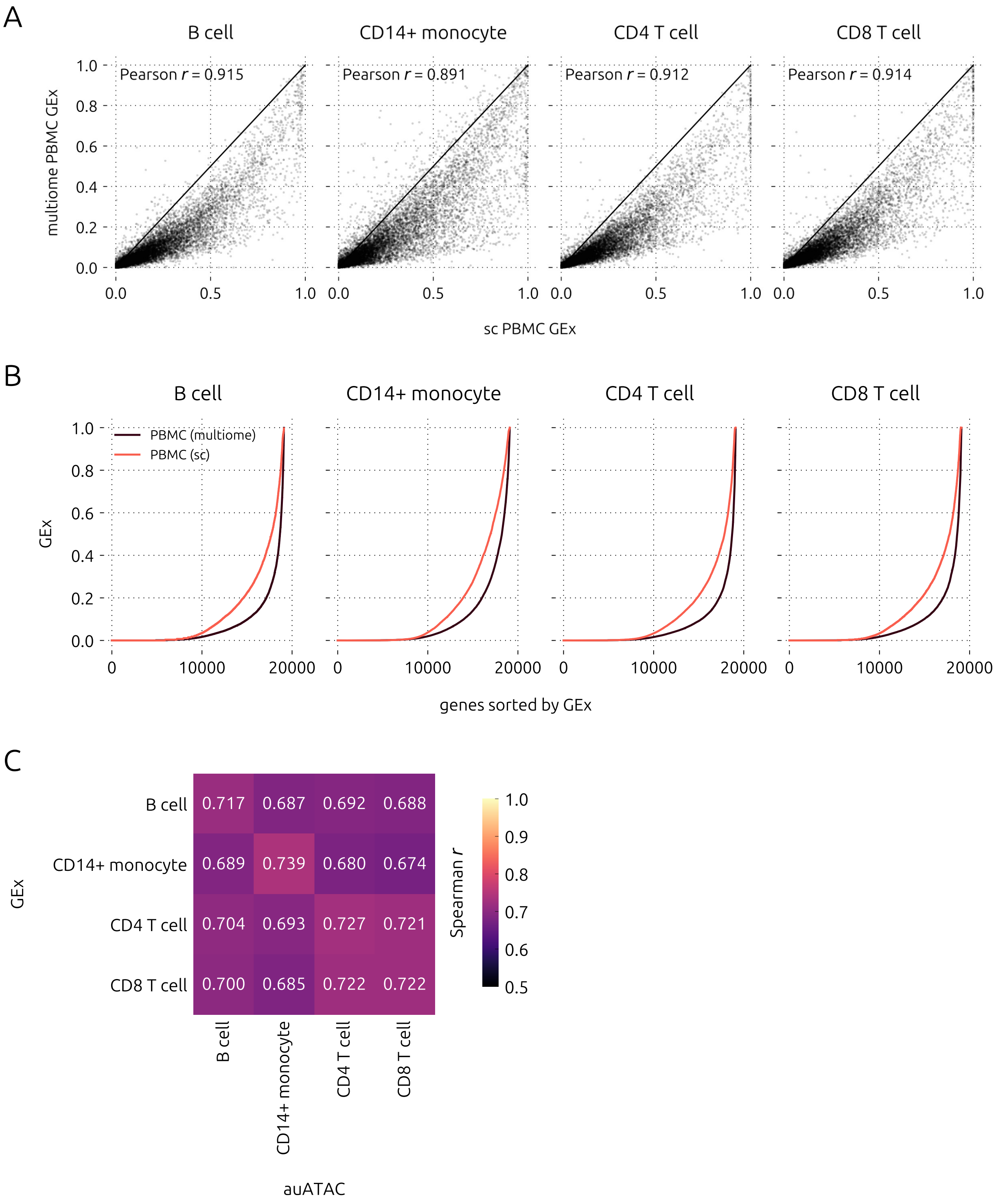

Supplementary Figure S11: Comparison of PBMC GEx between multiome and single-cell datasets. A. Multiome versus single-cell GEx in each cell type. B. Ranked GEx values in multiome and single-cell datasets. C. Spearman correlation between sc GEx and multiome auATAC for each major cell type.

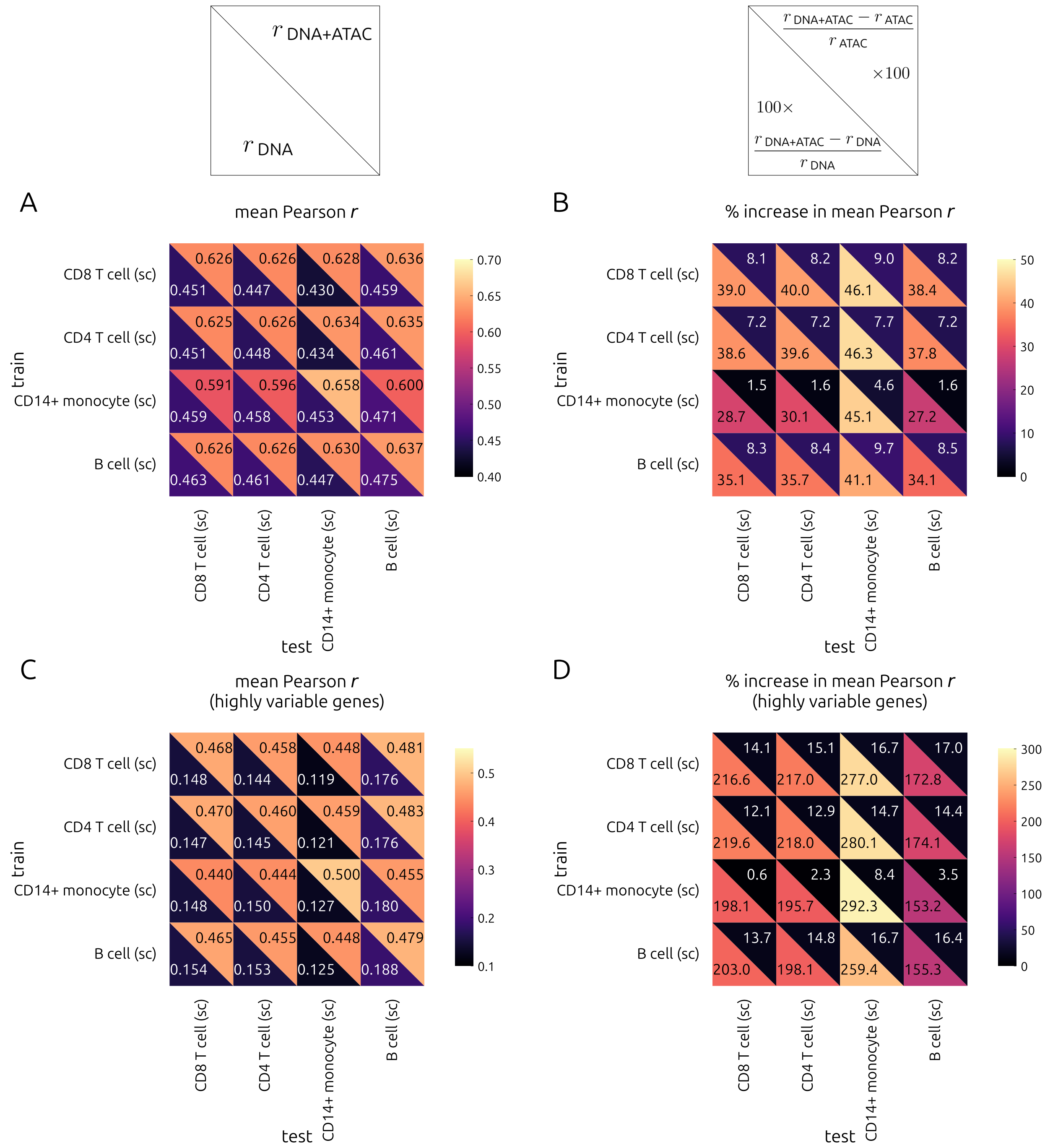

Supplementary Figure S12: Cross-cell type performance within the PBMC single-cell dataset, using ATAC data from the PBMC multiome dataset. A. Mean Pearson r of models trained on one cell type (rows) and tested on another (columns) for the PBMC single-cell dataset. Metrics for the DNA+ATAC model are shown in the top right triangle of each grid square. Metrics for the DNA-only model are shown in the bottom left triangle of each grid square. B. Same as A, but showing mean percent increase in Pearson r of the DNA+ATAC model relative to DNA only (bottom left) and ATAC only (top right). C. Same as A, but evaluated on highly variable genes. D. Same as B, but evaluated on highly variable genes.

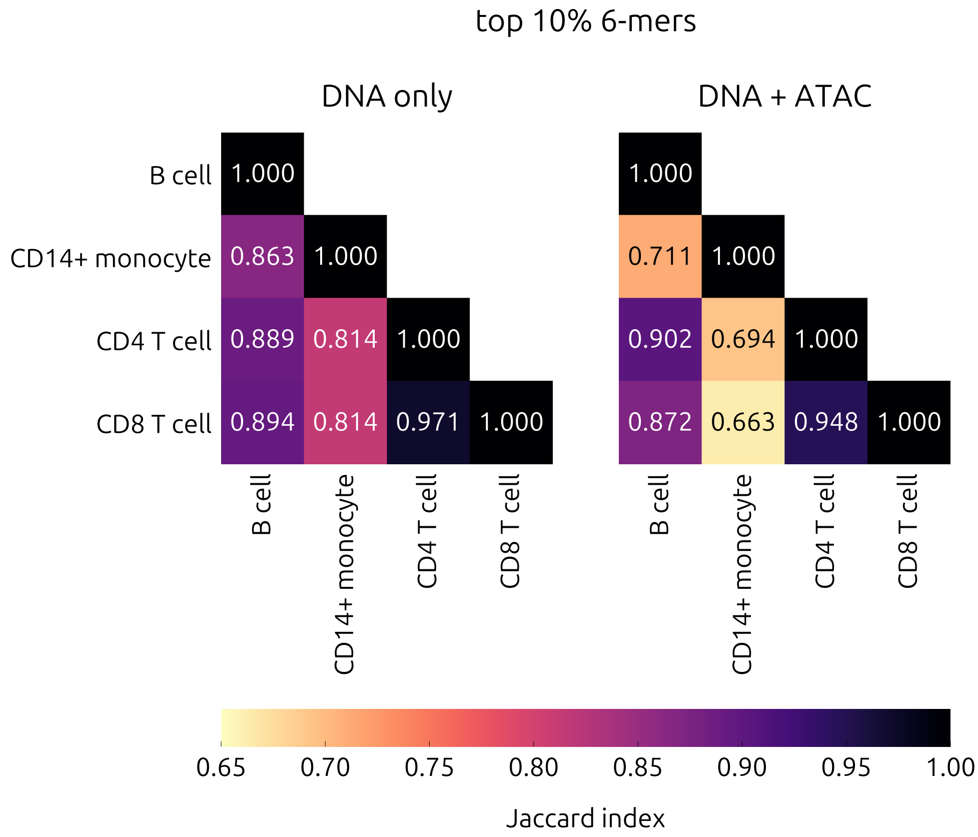

Supplementary Figure S13: Comparison of top 6-mers by mean attribution in the PBMC (sc) dataset, using the Jaccard index.

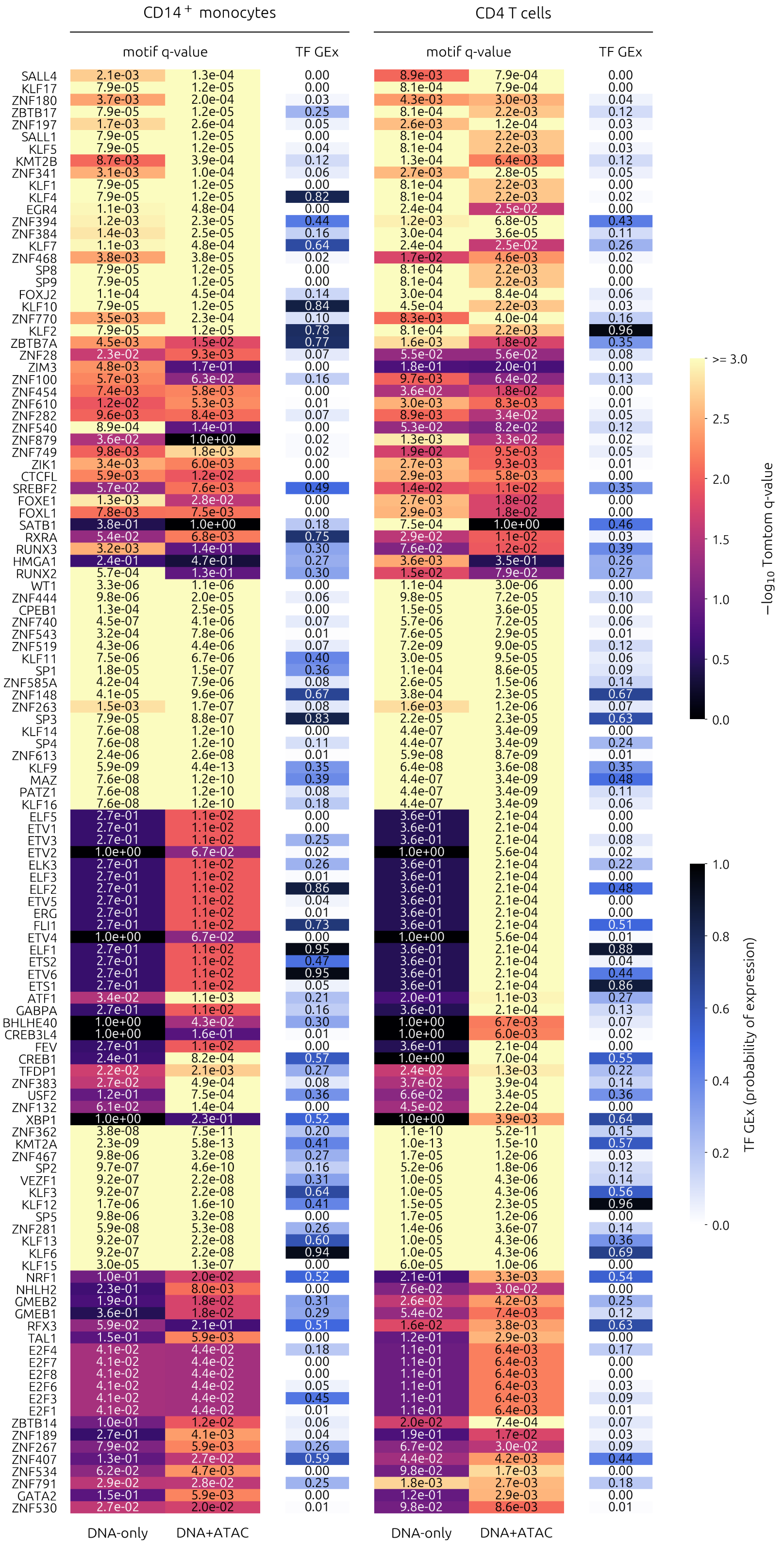

Supplementary Figure S14: Motifs discovered by TF-MoDISco for CD14$^+$ monocytes and CD4 T cells in the PBMC (sc) dataset. Motif q-values from Tomtom are shown, as well as the gene expression of the corresponding TF.

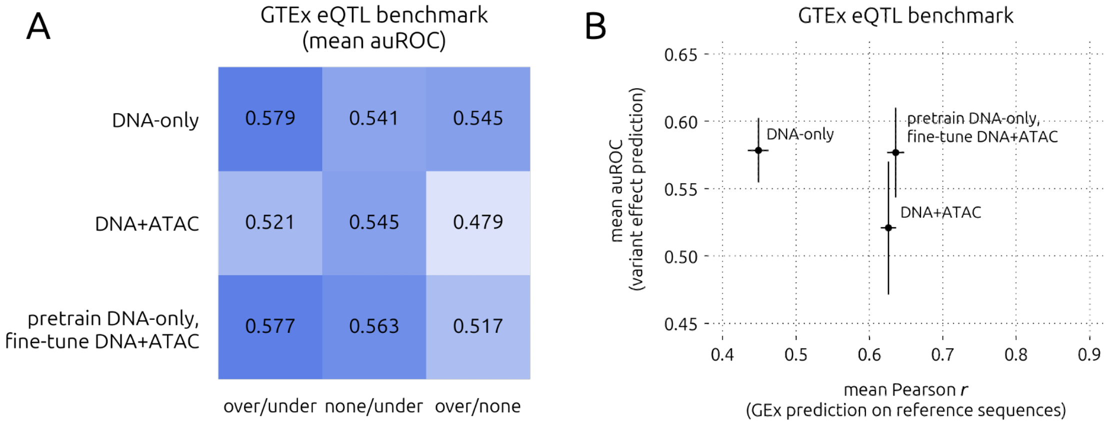

Supplementary Figure S15: Promoter variant effect prediction on the GTEx eQTL benchmark dataset curated by Jaganathan et al. (2025), using models trained on CD4 T cells (sc). A. Mean auROC across 5-fold CV of promoter variant effect classification on the held-out test genes. B. Mean auROC for over/under-expression classification plotted against mean model performance on the reference sequences with CD4 T cell (sc) GEx. Error bars show standard deviation.

References

K. Adelman and J. T. Lis. Promoter-proximal pausing of RNA polymerase II: emerging roles in metazoans. *Nature Reviews Genetics*, 13:720–731, 9 2012. doi: 10.1038/nrg3293.

V. Agarwal and D. R. Kelley. The genetic and biochemical determinants of mRNA degradation rates in mammals. *Genome Biology*, 23, 12 2022. doi: 10.1186/S13059-022-02811-X.

D. Bredikhin et al. MUON: multimodal omics analysis framework. *Genome Biology*, 23:1–12, 12 2022. doi: 10.1186/S13059-021-02577-8.

C. G. Danko et al. Dynamic evolution of regulatory element ensembles in primate CD4+ T cells. *Nature Ecology & Evolution*, 2:537–548, 1 2018. doi: 10.1038/s41559-017-0447-5.

A. Frankish et al. GENCODE: reference annotation for the human and mouse genomes in 2023. *Nucleic Acids Research*, 51(D1):D942–D949, 11 2022. doi: 10.1093/nar/gkac1071.

K. Jaganathan et al. Predicting expression-altering promoter mutations with deep learning. *Science*, 389(6760):eads7373, 2025. doi: 10.1126/science.ads7373.

M. Karlsson et al. A single–cell type transcriptomics map of human tissues. *Science Advances*, 7(31):eabh2169, 2021. doi: 10.1126/sciadv.abh2169.

L. Mcinnes et al. UMAP: Uniform manifold approximation and projection for dimension reduction, 2020.

M. P. Mulè et al. Normalizing and denoising protein expression data from droplet-based single cell profiling. *Nature Communications*, 13:1–12, 4 2022. doi: 10.1038/s41467-022-29356-8.

V. Satopa et al. Finding a "Kneedle" in a haystack: Detecting knee points in system behavior. In *International Conference on Distributed Computing Systems Workshops*, pages 166–171, Minneapolis, MN, USA, 2011.

V. A. Traag et al. From louvain to leiden: guaranteeing well-connected communities. *Scientific Reports*, 9, 12 2019. doi: 10.1038/S41598-019-41695-Z.

M. Uhlen et al. A genome-wide transcriptomic analysis of protein-coding genes in human blood cells. *Science*, 366(6472):eaax9198, 2019. doi: 10.1126/science.aax9198.

F. A. Wolf et al. Scanpy: Large-scale single-cell gene expression data analysis. *Genome Biology*, 19:1–5, 2 2018. doi: 10.1186/S13059-017-1382-0.
